# Tidying-up the plant nuclear space: domains, functions, and dynamics

**DOI:** 10.1093/jxb/eraa282

**Published:** 2020-06-18

**Authors:** Ana Paula Santos, Valérie Gaudin, Iva Mozgová, Frédéric Pontvianne, Daniel Schubert, Ahmet L Tek, Martina Dvořáčková, Chang Liu, Paul Fransz, Stefanie Rosa, Sara Farrona

**Affiliations:** 1 Instituto de Tecnologia Química e Biológica António Xavier, Universidade Nova de Lisboa, Oeiras, Portugal; 2 Institut Jean-Pierre Bourgin, INRAE, AgroParisTech, Université Paris-Saclay, Versailles, France; 3 Biology Centre of the Czech Academy of Sciences, České Budějovice, Czech Republic; 4 Faculty of Science, University of South Bohemia, České Budějovice, Czech Republic; 5 CNRS, Laboratoire Génome et Développement des Plantes (LGDP), Université de Perpignan Via Domitia, Perpignan, France; 6 Institute for Biology, Freie Universität Berlin, Berlin, Germany; 7 Agricultural Genetic Engineering Department, Niğde Ömer Halisdemir University, Niğde, Turkey; 8 CEITEC/Masaryk University, Brno, Czech Republic; 9 Center for Plant Molecular Biology (ZMBP), University of Tübingen, Tübingen, Germany; 10 Institute of Biology, University of Hohenheim, Stuttgart, Germany; 11 University of Amsterdam, Amsterdam, The Netherlands; 12 Swedish University of Agricultural Sciences, Uppsala, Sweden; 13 Plant and AgriBiosciences Centre, Ryan Institute, NUI Galway, Galway, Ireland; 14 Universidad Complutense de Madrid, Spain

**Keywords:** 3D Chromatin organization, chromocentres, gene expression, liquid–liquid phase separation (LLPS), nuclear domains, nuclear bodies, nucleolus, nuclear periphery, telomeres, topologically associated domains (TADs)

## Abstract

Understanding how the packaging of chromatin in the nucleus is regulated and organized to guide complex cellular and developmental programmes, as well as responses to environmental cues is a major question in biology. Technological advances have allowed remarkable progress within this field over the last years. However, we still know very little about how the 3D genome organization within the cell nucleus contributes to the regulation of gene expression. The nuclear space is compartmentalized in several domains such as the nucleolus, chromocentres, telomeres, protein bodies, and the nuclear periphery without the presence of a membrane around these domains. The role of these domains and their possible impact on nuclear activities is currently under intense investigation. In this review, we discuss new data from research in plants that clarify functional links between the organization of different nuclear domains and plant genome function with an emphasis on the potential of this organization for gene regulation.

## Teaser

We review recent discoveries that have elucidated how the spatial organization of the nuclear DNA in functional domains is relevant for the regulation of the genome in plants.

## Introduction

**Fig. 1.**
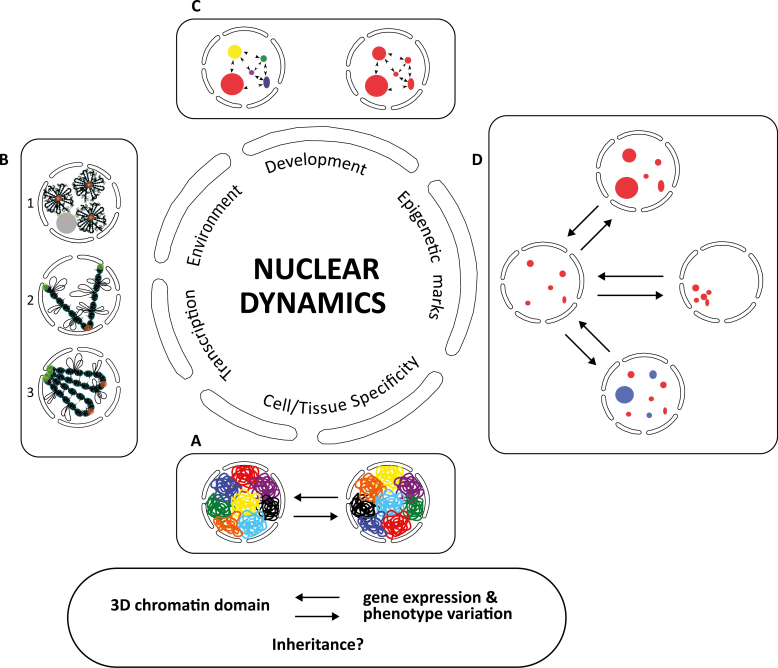
3D Nuclear organization and dynamics. Schematic representation of chromatin plasticity subjacent to transcriptional requirements, environmental responses, cell and tissue specificities, developmental stages, and epigenetic marks. (A) Flexible organization of chromosome territories. (B) Flexible positioning and organization of genes, chromocentres (red dots), and telomeres (green dots) inherent to distinct models of chromosome configurations: rosette-like configuration (1); Rabl configuration (2); and bouquet configuration (3). (C) Possible crosstalk between different nuclear domains may occur as part of nuclear dynamics (reviewed in Kumaran *et al.*, 2008). (D) Number, size, formation/disappearance, and organization of nuclear domains may change in response to different stimuli.

In eukaryotic cells, the genetic information is encoded by DNA, which can be several metres long, that needs to be packaged to fit into the cell nucleus. However, the function of the nucleus goes much further than just being a simple packaging entity. The three-dimensional (3D) organization of the interphase nucleus remained unknown for a long time and only with substantial improvements in microscope resolution and *in situ* staining techniques was it possible to visualize distinct chromatin domains and chromosome territories in the interphase nucleus (Fig. 1) (Cremer and Cremer, 2001, 2010). Historically, insights into the position and organization of chromatin domains and chromosomes within the plant cell nucleus come from visual approaches, such as fluorescence *in situ* hybridization (FISH) and genomic *in situ* hybridization (GISH) (reviewed in Santos *et al.*, 2015). In plants, the observation of chromosome territorial organization was not straightforward mainly due to the complexity of plant genomes with a high amount of dispersed repetitive sequences and the insufficient signal intensity of short unique sequences which hampered the understanding of plant territorial chromatin organization. Pioneer studies involving the ingenious implementation of GISH in interspecific and intergeneric hybrids (e.g. wheat/rye translocation or addition lines) made possible the visualization of chromosome territories in plant cell nuclei (Fig. 1) (Schwarzacher *et al.*, 1992; Aragon-Alcaide *et al.*, 1997; Bass *et al.*, 2000). These studies were based on labelling introgressed chromatin and only later was a ‘true chromosome painting’ done in *Arabidopsis thaliana* (Arabidopsis) and its close relative *A. lyrata* by using chromosome-specific probes (Fransz *et al.*, 2002). More recently, the use of synthetic oligonucleotide libraries enabled whole-chromosome oligo-FISH paints of maize chromosomes to be obtained (Albert *et al.*, 2019).

The higher order organization level of chromatin into functional nuclear domains has the potential to deeply affect gene regulation (reviewed in Adriaens *et al.*, 2018; Groves *et al.*, 2018). Some nuclear domains such as the nucleolus and large-scale heterochromatin foci (chromocentres) are well recognized by phase contrast microscopy due to differential DNA concentration or protein density. Other domains, such as nuclear bodies (NBs) enriched in regulatory proteins [e.g. Cajal bodies (CBs) or Polycomb bodies (PcBs)], can only be detected by immunostaining with domain-specific antibodies. In addition, the nuclear envelope (NE) and the underlying lamina have their own space and organization within the 3D nuclear structure. All nuclear subdomains have in common the lack of a membrane structure confining their space, yet each NB forms a discrete nuclear compartment exerting a specific function. Other nuclear structures, such as the telomeres, despite not being bona fide subdomains, also have a demonstrated special organization and function within the nucleus.

Furthermore, innovative approaches based on large-scale chromosomal capture approaches, in combination with deep profiling and high-throughput sequencing, make it feasible to annotate topologically associated domains (TADs) within the chromatin and even to identify TADs from a more specific perspective, namely nucleolar-associated domains (NADs) and lamina-associated domains (LADs) (Dixon *et al.*, 2012; Feng *et al.*, 2014; reviewed in Nicodemi and Pombo, 2014; Ciabrelli and Cavalli, 2015; Pontvianne and Grob, 2020). Moreover, chromatin domains often show specific epigenetic modification patterns and transcriptional states, suggesting that the higher level of chromatin regulation does entail a functional role. It remains a challenge to understand the mechanisms assisting chromatin organization and the extent of chromatin flexibility in response to specific transcriptional requirements or environmental stress conditions. The exposure to stress factors can trigger changes in large-scale genome organization including rearrangements of chromatin structure and spatial nuclear organization (reviewed in Probst and Mittelsten Scheid, 2015; Santos *et al.*, 2017) as predicted by McClintock (1984). Still much of the evidence has been gained from studies in metazoans and much less attention has been given to the details of nuclear organization in plants. While there are several organizing principles partially conserved between plants and animals, recent studies have started to provide details regarding specificities of plant nuclear organization. For instance, plants show several exclusive chromatin-associated characteristics, including plant-specific histone variants (reviewed in Zambrano-Mila *et al.*, 2019), novel DNA- and histone-modifying enzymes, plant-specific histone modifications (reviewed in Feng and Jacobsen, 2011), as well as a plant-specific lamina-like network at the nuclear periphery (Poulet *et al.*, 2017*b*). These specificities of chromatin organization and epigenetics make plants a very interesting model to study the plasticity of nuclear dynamics.

Our aim here is to give an overview of the different nuclear domains in plants, as well as to provide some insights into how they may be organized without the requirement of a membrane to compartmentalize them from surrounding domains and how they may impact gene expression.

## Nuclear bodies

Originally detected as cytological structures, NBs are membrane-less functional compartments interspersed in the nucleoplasm, that are now recognized to participate in the spatiotemporal control of various specialized nuclear processes (i.e. transcription, RNA processing, DNA replication, DNA repair, protein degradation, and signal transduction) (reviewed in Del Prete *et al.*, 2014; Guo and Fang, 2014; Staněk and Fox, 2017; Shah *et al.*, 2018). Various names are commonly used for NBs, such as foci, speckles, or paraspeckles—these terms are usually not clearly defined and are based on original cytological observations before functional elements were identified, and sometimes may refer to the size or organization of the observed pattern. Most of them are dynamic structures depending on physiological, developmental, or stress conditions (Fig. 1) (Boudonck *et al.*, 1999; reviewed in Reddy *et al.*, 2012; Scarpin *et al.*, 2013; Kim *et al.*, 2019). Beyond their diversity, a common feature of NBs is their role in the formation of specific microenvironments, characterized by supramolecular assembly of proteins, in some cases accompanied by RNA molecules. The local concentration of NB components contributes to the molecular crowding-increased binding rate and low diffusion rate, which favour biochemical reactions (reviewed in Zhu and Brangwynne, 2015). Thus, NBs are centres for enzymatic reactions but also sequestration, storage, modification, recycling, or degradation of proteins. Some of them play a hub role in scaffolding and recruiting genomic regions with similar regulation, thus participating in the 3D genome organization. The nucleolus, as the largest NB, is discussed in the following section of this review.

PcBs, first described in animal cells, are marked by local accumulation of Polycomb-group (PcG) proteins. PcBs concentrate distant transcriptionally inactive PcG-targeted genomic regions that form intra- or interchromosomal interactions also detectable by chromatin conformation capture techniques (reviewed in Matheson and Elderkin, 2018; Loubiere *et al.*, 2019). Unlike in animals, PcG-targeted regions are more dispersed throughout the plant genome (reviewed in Del Prete *et al.*, 2015). Nevertheless, genomic regions targeted by the Arabidopsis PcG protein LIKE HETEROCHROMATIN PROTEIN 1 (LHP1) tend to be organized locally in clusters along the chromosomes (Molitor *et al.*, 2016). In the nuclear space, LHP1 and several Arabidopsis PcG proteins form foci when transiently overexpressed in heterologous systems [LHP1 (Gaudin *et al.*, 2001), CURLY LEAF (CLF) (Hohenstatt *et al.*, 2018), VERNALIZATION 2 (VRN2) (Gendall *et al.*, 2001), and EMBRYONIC FLOWER 1 (EMF1) (Calonje *et al.*, 2008)]. Such foci were also observed in complemented conditions for LHP1 (Kotake *et al.*, 2003) whose pattern is dependent on cell differentiation (Libault *et al.*, 2005), similarly to animal PcGs (Ren *et al.*, 2008; Kundu *et al.*, 2017). A non-uniform nuclear distribution is adopted by the protein PWWP-DOMAIN INTERACTOR OF POLYCOMBS1 (PWO1) under conditions of native promoter-driven expression in Arabidopsis (Mikulski *et al.*, 2019). Finally, a physical clustering of repressed alleles of the Polycomb Repressive Complex (PRC) 2 target *FLOWERING LOCUS C* (*FLC*) was reported in Arabidopsis interphase nuclei, which relies on PHD–PRC2 components (Rosa *et al.*, 2013). These data show that clustering of PcG proteins and their genomic targets also occurs in plants, implying that PcBs are likely to be common features of animal and plant nuclei. The functional implications of PcBs and compact domains of PcG-targeted genes in PcG repression or other processes remain debated in animals (reviewed in Matheson and Elderkin, 2018), and further investigations are required in plants. MORC (Microrchidia) family ATPase-enriched NBs appear to be another type of Arabidopsis NB associated with silencing, implicated in repression of DNA-methylated pericentromeric genes (Moissiard *et al.*, 2012) or unmethylated pathogen-responsive genes (Harris *et al.*, 2016).

Components of the transcription machinery in animals were originally described as concentrated aggregates within the nucleus and were named transcription factories (TrFs) (Buckley and Lis, 2014). A dispersed and reticulated distribution of RNA polymerase II was reported in Arabidopsis (Schubert and Weisshart, 2015). TrF definition may evolve with microscopy and live imaging development (Buckley and Lis, 2014). TrFs were recently proposed in wheat (Concia *et al.*, 2020).

Cajal bodies (CBs, or coiled bodies) are dynamic substructures, which are found in animal as well as plant cells (Boudonck *et al.*, 1998, 1999; reviewed in Gall, 2000). CBs are enriched for splicing components such as small nuclear ribonucleoproteins (snRNPs) and are usually associated with the conserved coilin (Collier *et al.*, 2006; reviewed in Machyna *et al.*, 2015), both being structural scaffolds for CB formation (reviewed in Bassett, 2012; Staněk and Fox, 2017; Ohtani, 2017). CBs are involved in maturation of spliceosome snRNPs, snRNA chemical modifications, and RNP export complex assembly. Recent data support a role for CBs in the topological organization of spliceosomal snRNA and histone genes in inter- and intrachromosomal gene clusters and in their transcriptional regulation ( Sawyer *et al.*, 2016; Wang *et al.*, 2016). In addition, CBs contribute to telomere maintenance, ribosome biogenesis, or stress responses via poly(ADP ribose) polymerase interactions (Kotova *et al.*, 2009; reviewed in Boulon *et al.*, 2010; Love *et al.*, 2017). Plant CBs have additional functions in siRNA and miRNA processing (Pontes and Pikaard, 2008; Scarpin *et al.*, 2013; Shaw *et al.*, 2014). In plants, not all snRNP-containing bodies resembling CBs in size and shape are associated with the presence of coilin and it remains debated whether these should be classified as CBs (Pontes and Pikaard, 2008; Pontes *et al.*, 2013; Love *et al.*, 2017). Among them are splicing speckles (SpSs) and nuclear dicing bodies (D-bodies). SpSs are general sites of storage and assembly of splicing regulators in regions of active transcription, and are conserved in animals and plants. In addition to splicing factors, they also concentrate RNA polymerase II subunits, transcription elongation and polyadenylation factors, and chromatin proteins, suggesting broader functions (reviewed in Galganski *et al.*, 2017). Located in interchromatin regions, SpSs are dynamic and mobile depending on transcriptional activity, cell differentiation, or metabolic state (Docquier *et al.*, 2004; Kim *et al.*, 2019; for reviews see Reddy *et al.*, 2012; Galganski *et al.*, 2017). D-bodies are plant-specific NBs that are centres of pri-miRNA processing and contain microprocessor complex components including DICER-LIKE 1 or HYPONASTIC LEAVES 1 (Fujioka *et al.*, 2007; Fang and Spector, 2007; reviewed in Dolata *et al.*, 2018).

NBs can also form upon environmental or developmental stimuli. In plant nuclei, DNA damage and repair proteins form distinct foci upon DNA damage that are dynamic (Friesner *et al.*, 2005). The RAD54-marked foci, for instance, tend to locate at the nuclear periphery during DNA repair (Hirakawa and Matsunaga, 2019). Recently, antiviral immunity-related NBs were also found in plants, such as NBs formed with the WW-domain protein AtWWP1, which sequester viral nucleoprotein complexes in the nucleus and prevent their nucleoplasmic trafficking (Calil *et al.*, 2018). The plant-specific photobodies (PBs) are enriched in photoreceptors, light signalling, and proteasome degradation components, transcription regulators, or splicing factors that are involved in light-induced photoreceptor sequestration, photomorphogenesis inhibitor protein degradation, and light-responsive transcript processing (Bauer *et al.*, 2004; Galvão *et al.*, 2012; Kaiserli *et al.*, 2015; Xin *et al.*, 2017, 2019). PB assembly and dynamics correlate with plant growth and developmental processes in response to light signals (reviewed in Chen and Chory, 2011; van Buskirk *et al.*, 2012; Klose *et al.*, 2015). PB biogenesis depends on light signalling component interactions which can individually nucleate *de novo* formation of the PBs (Liu *et al.*, 2014). Recently, several novel factors required for the formation of PHY-B-containing PBs have been identified (Qiu *et al.*, 2017; H. Huang *et al.*, 2019; Yoo *et al.*, 2019). Phytohormone-induced NBs were reported, but remain poorly characterized (Ng *et al.*, 2004; Riera *et al.*, 2006).

Despite significant progress, many questions remain to be addressed regarding plant NB structure and functions, which may reveal originalities, connected for instance to the different subcellular sequestration of processes conserved in plants and animals, such as siRNA/miRNA processing or nonsense-mediated decay (reviewed in Pontes and Pikaard, 2008). Specific features of plant NBs such as higher plasticity and/or redundancy of NB components are also hinted at by the absence of phenotype effects upon depletion of coilin (Collier *et al.*, 2006) despite drastic effects of its depletion in animals (Walker *et al.*, 2009). With the development of high-resolution microscopy tools as well as biochemical approaches, the composition, classification, and substructure of plant and animal NBs are expected to evolve with expansion of the NB repertoire and redefinitions of NBs in terms of detailed structural or functional qualities. For instance, besides the internal nucleolar organization, subcompartmentalization has been reported for smaller NBs such as paraspeckles and speckles (Mintz and Spector, 2000; Hall *et al.*, 2006; West *et al.*, 2016; Fei *et al.*, 2017). This highlights a higher level of structural complexity of the cell nucleus that may account for the multitude of functions associated with certain NB types and opens up further questions regarding NB substructure dynamics. Improvement of live imaging and tracking techniques is expected to shed new light on NB distribution, mobility, and function. Recently, long-range, directional, actin-independent motion of speckles within chromatin-depleted channels highlighted a novel nuclear trafficking mechanism (Kim *et al.*, 2019). A novel technique allowing identification of NB-associated genomic regions (Baudement *et al.*, 2018) can help in addressing the interplay between the NB structure and 3D genome organization.

How NBs are assembled is a challenging interdisciplinary question addressed by cellular and structural biologists, biophysicists, and modellers. Assembly models are currently proposed. The first model is based on a sequential and ordered assembly of the NB components anchored to major scaffolding NB proteins or RNA molecules. The second model favours stochastic processes as contributing to the random assembly of different components in a self-organizing manner, which can start with any NB component (reviewed in Matera *et al.*, 2009; Mao *et al.*, 2011). However, the observed non-random organizations suggest more complex rules and question the physicochemical forces driving NB assembly.

## Chromocentres

In a chromosome, the centromeres together with the nearby chromatin regions can form heterochromatin structures that remain condensed during interphase (Heitz, 1931). In Arabidopsis, the term chromocentres refers to centromeric and pericentromeric regions forming heterochromatic domains during interphase (Fransz *et al.*, 2002). In other species, such as *Drosophila*, the term chromocentres originally and more formally refers to the congregation of pericentromeric heterochromatic regions from different chromosomes forming a small number of chromocentres (Jagannathan *et al.*, 2019). In maize, heterochomatic knobs are distal, satellite tandem repeats, not centromeric, and fit the ‘staining’ definition of chromocentres, but are not called chromocentres because they are not aggregations of centric heterochromatin (reviewed in Gent *et al.*, 2017). In general, centromeres are enriched in satellite repeats and retrotransposons (Nagaki *et al.*, 2003; Tek *et al.*, 2010; reviewed in Malik and Henikoff, 2009).

Cytologically, DNA staining intensity by DAPI has been used to visualize chromocentres which are generally round shaped, highly condensed, and heavily stained prominent structures (Fransz *et al.*, 2002; Tek *et al.*, 2011). Usually the boundaries of actual centromeres and pericentromeric regions within the chromocentres are not clearly defined, although in well-studied plants such as Arabidopsis this distinction has already been made (reviewed in Simon *et al.*, 2015). Specifically, in Arabidopsis, within the whole array of 180 bp centromeric DNA repeats, only a limited portion is recognized by immunolabelling with HTR12 protein, a homologue of the centromere-specific histone H3 variant (CENH3) (Shibata and Murata, 2004).

Although the DNA composition of chromocentres is broadly known, their function remains elusive (reviewed in Simon *et al.*, 2015; Jagannathan and Yamashita, 2017). More recently, new models of possible functions have proposed that chromocentre formation could be involved in the maintenance of the eukaryotic genome (reviewed in Jagannathan and Yamashita, 2017). Nevertheless, because of their tractability, chromocentres have largely been used as targets to study genome structure and organization. In Arabidopsis, the chromocentres tend to be preferentially located at the nuclear periphery (Fransz *et al.*, 2002; Pecinka *et al.*, 2004). It is from the chromocentres that chromatin loops (0.2–2 Mb in length) emanate, giving rise to a rosette-like interphase chromosome configuration (Fransz *et al.*, 2002) (Fig. 1). Contrastingly, in larger genomes such as wheat, chromocentres were not described, and centromeres were visualized by FISH as being polarized on one side of the nucleus to establish a special organization known as the Rabl configuration of interphase chromosomes (Santos and Shaw, 2004) (Fig. 1). These studies illustrate that centromere positioning in mitotic anaphase results in establishing the polarized Rabl nuclear arrangement, thereby affording centromeres more opportunities for interaction than would occur if chromosomes were randomly distributed. The morphological variability of chromocentres present in different Arabidopsis ecotypes has also been used to determine the genetic factors governing chromocentre formation and maintenance, as it was possible to determine several genetic loci affecting chromocentre structure. These results indicate the involvement of complex genetic mechanisms in chromatin organization (Snoek *et al.*, 2017).

Chromocentres exhibit epigenetically distinct chromatin features. The most obvious property of chromocentres is their heavy DNA methylation, as evidenced by immunolabelling with 5-methylcytosine antibodies (Fransz *et al.*, 2002). Also H3K9me2 levels were increased at Arabidopsis chromocentres (Zhang *et al.*, 2008). On the other hand, histone marks related to transcriptionally active chromatin, such as the acetylation of histone H4 (H4K5ac and H4K8ac), were not detected at chromocentres (Fransz *et al.*, 2002), reinforcing the idea of transcriptional inactivity of chromocentres in Arabidopsis. Indeed, chromocentre DNA sequences are transcriptionally repressed, forming silenced domains (Soppe *et al.*, 2002), and transcription of transposable elements in the chromocentres is reduced during *de novo* chromatin formation (Benoit *et al.*, 2019).

Environmental factors are known to affect the chromatin structure (reviewed in Probst and Mittelsten Scheid, 2015), and chromocentres have in fact been used as target sites in the nuclei to track the effect of environmental stresses. Imposed heat stress on Arabidopsis caused decondensation of the chromocentre structure with an increase in transcriptional activation of repetitive elements but without any change in DNA methylation (Pecinka *et al.*, 2010). HEAT-INTOLERANT 4 (HIT4) orchestrates heat-mediated chromocentre decondensation and subsequent activation of centromeric sequences (Wang *et al.*, 2013). As a negative regulator of gene expression under various stress conditions, STRESS RESPONSE SUPPRESSOR1 (STRS1), a DEAD-box RNA helicase involved in RNA metabolism, is located at the chromocentres but leaves the chromocentres in response to salt stress, possibly contributing to gene silencing via an interacting partner protein (Khan *et al.*, 2014). The environmental-mediated changes in chromocentric structure are therefore a very good indicator of the dynamism of this nuclear structure as well as of the chromocentre-associated proteins and of the transcriptional regulation of chromocentre-localized sequences. This dynamic response in chromocentre organization has also been shown during development. For instance, in Arabidopsis, the non-random arrangement of chromocentres in the diploid cell and also in the triploid endosperm implicates specific interactions of parental chromosomes for the developmental-mediated epigenetic mechanisms during seed development (Baroux *et al.*, 2017). An increased number of chromocentres is correlated with dosage of maternal chromosomes, indicating the requirement for the separation of maternal chromosomes (Baroux *et al.*, 2017). Clearly, defined features of chromocentres in Arabidopsis along with variable developmental stages and environmental stimuli could pave the way for refinement of genetic determinants affecting the chromatin organization and its effect in gene regulation. Formation of chromocentres during developmental stages requires highly regulated changes in nucleosome structure and histone post-translational modifications (Benoit *et al.*, 2019).

Satellite DNA sequences have been used to track the chromocentres in Arabidopsis, for example by FISH and zinc finger DNA recognition coupled to green fluorescent protein (GFP) (Lindhout *et al.*, 2007). The development of tracking methods for the chromocentres using fluorescent transcription activator-like effectors (TALEs) (Fujimoto *et al.*, 2016) or fluorescent versions of the CRISPR/Cas [clustered regularly interspaced palindromic repeats (CRISPR)/CRISPR-associated protein] system (Anton *et al.*, 2014) in live tissues while retaining the morphology will provide novel insights in the analysis of chromatin dynamics during variable environmental and developmental conditions. These, together with other tools, such as GFP-tagged CENH3 (De Storme *et al.*, 2016), will hopefully broaden the current understanding of chromocentre properties and functions in a variety of cells and tissues.

## The nucleolus

Our knowledge on this nuclear compartment goes back to the 18th century, when it was first observed and reported by Felice Fontana, who noticed its occurrence in the slime of an eel (reviewed in Pederson, 2011). In 1839, Gabriel Gustav Valentin described this structure as a ‘nucleus within the nucleus’ and named it ‘nucleolus’. In the 1930s, the link between the nucleolus and ribosome biogenesis was established by other leading scientists. In 1931, the cytogeneticist Heitz described the presence of a secondary constriction on some chromosomes, different from the centromere, which Barbara McClintock correlated with the nucleolus in corn (Heitz, 1931; McClintock, 1934). Barbara McClintock designated the secondary constrictions as ‘NORs’ for ‘nucleolus organizer regions’, without knowing the content of these genomic regions. The direct link between NORs, ribosome biogenesis, and the nucleolus was then clearly established in the 1960s (Brown and Gurdon, 1964; Ritossa and Spiegelman, 1965; Warner and Soeiro, 1967). The structure of the nucleolus, which is the consequence of ribosome biogenesis, is divided into three different subcompartments: the fibrillar compartment (FC), surrounded by the dense fibrillar component (DFC), and the granular component (GC). These subcompartments correspond to different phases of the ribosome biogenesis process, starting from the transcription of rRNA precursor by RNA polymerase I at the FC/DFC boundary, to the formation of the pre-ribosome complexes in both DFC and then GC compartments (reviewed in Stępiński, 2014). Hundreds of factors and steps are required to generate mature ribosome subunits, and current knowledge has been recently reviewed in Sáez-Vásquez and Delseny (2019). Although formation of the nucleolus is a direct consequence of ribosome biogenesis, additional functions have been linked to the nucleolus and include cell growth regulation, stress response, cell ageing, ribonucleoprotein complex formation, RNA degradation, and genome organization (reviewed in Boisvert *et al.*, 2007; Boulon *et al.*, 2010).

In plant cells, a first proteomic analysis identified >200 nucleolar proteins, mainly implicated in ribosome biogenesis, as well as in RNA metabolism (Pendle *et al.*, 2005). More recent reports almost doubled this number and revealed an additional link with the proteasome (Palm *et al.*, 2016; Montacié *et al.*, 2017). At the genomic level, although the presence of rRNA genes was clearly established, additional works revealed that both active and inactive rRNA genes co-exist in every cell (reviewed in Grummt and Pikaard, 2003). However, only active rRNA genes are present within the nucleolus, as demonstrated by the analysis of purified nucleoli of Arabidopsis (Pontvianne *et al.*, 2013). Expressed rDNA copies associate with active chromatin marks: cytosines are poorly methylated in every context and histones are mainly methylated at Lys4 and Lys36 of the histone 3 (H3K4me3 and H3K36me3, respectively). Conversely, silent copies remain excluded from the interior of the nucleolus and contain constitutive heterochromatin marks such as H3K9me2 and H3K27me1 (Lawrence *et al.*, 2004; Pontvianne *et al.*, 2012, 2013).

**Fig. 2.**
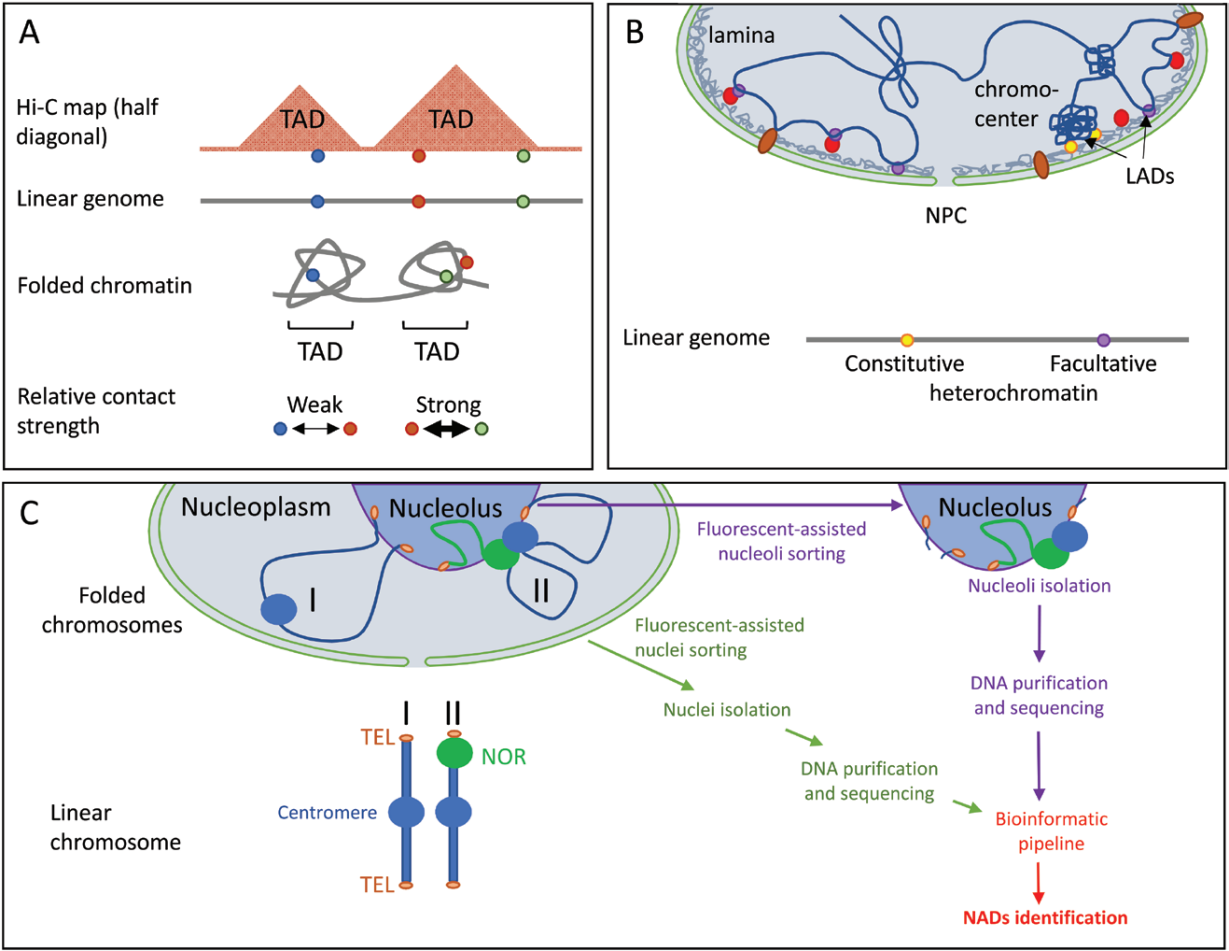
Schematic illustration of TADs, NADs, and LADs. (A) Self-organized chromatin domains and their corresponding TAD patterns on a Hi-C map. In this sketch, three evenly distributed genomic loci are distributed in two TADs; the two located in the same TAD show stronger chromatin contact (shorter physical distance) than loci in different TADs. (B) Self-organized LADs, at either H3K9me2-marked constitutive heterochromatin (mainly at chromocentres) or H3K27me3-marked facultative heterochromatin. Association of chromatin at LADs is either directly with structural lamina components (grey mesh) or with lamina-interacting proteins (red and brown circles). (C) Self-organized chromatin domains associating with the nucleolus. In this sketch, two chromosomes are presented: one without an NOR (I) and one with an NOR (II). Chromosome (I) mainly associates with the nucleolus at its subtelomeric regions, while the NOR-bearing chromosome (II) shows stronger nucleolar association. For NAD identification, nuclei and nucleoli are isolated by fluorescent-assisted cell sorting. Nuclear and nucleolar DNA are then purified and sequenced to perform NAD identification.

In 2010, large chromatin domains, other than active NORs, were shown to associate with the nucleolus in mammalian cells (Németh *et al.*, 2010; Van Koningsbruggen *et al.*, 2010) and were named nucleolus-associated chromatin domains (NADs). NADs were also identified in Arabidopsis after isolation of nucleoli by fluorescent-assisted nucleoli sorting (FANoS) (Pontvianne *et al.*, 2016*a*; Carpentier *et al.*, 2018). As in mammalian cells, NADs in plants are primarily gene-poor regions enriched in repetitive sequences (reviewed in Picart-Picolo *et al.*, 2019; Pontvianne and Grob, 2020). In Arabidopsis, excluding rRNA genes, NADs represent 4% of the genome. At the chromosomal scale, most of the NADs are found on the short arm and at the subtelomeric regions of chromosome 4 (Pontvianne *et al.*, 2016*b*). These locations correspond to genomic regions flanking active rRNA genes located in the NOR on chromosome 4, as well as the telomeres that cluster at the nucleolar periphery in Arabidopsis, as will be further discussed (Fransz *et al.*, 2002; Chandrasekhara *et al.*, 2016) (Fig. 2). Little overlap can be found between NADs and genomic regions present at the nuclear periphery (i.e. LADs; see below) (reviewed in Picart-Picolo *et al.*, 2019; Pontvianne and Liu, 2020; Pontvianne and Grob, 2020). Interestingly, in the Arabidopsis *nucleolin 1* mutant, where both rRNA gene expression and nucleolar structure are altered, additional genomic regions that juxtapose the NOR2 on chromosome 2 now associate with the nucleolus. In addition, telomere nucleolar clustering is affected and telomeres are shorter (Pontvianne *et al.*, 2007, 2016*b*; reviewed in Picart and Pontvianne, 2017).

Around 900 genes localizing in the NADs or NAD genes have been identified in Arabidopsis leaf cells. Most of them are poorly expressed genes, and only pseudogenes and tRNA genes have been shown to be enriched in NADs (Pontvianne *et al.*, 2016*b*). It is important to note that RNA polymerase II is absent from the nucleolus (Schubert and Weisshart, 2015). The nucleolus could therefore be considered as a sequestering area in order to maintain certain genes silent. In mammals, actively transcribed regions are excluded from the nucleolar compartment, supporting the idea that the positioning close to the nucleolus is mainly linked to repressive states (Quinodoz *et al.*, 2018). In human cells, acidosis or heat shock stress provokes the retention of particular nucleoplasmic proteins within the nucleolus (Audas *et al.*, 2012; Jacob *et al.*, 2013). This phenomenon, known as stress-induced sequestration in the nucleolus, depends on production of long non-coding RNA from the intergenic regions of rRNA genes (reviewed in Audas and Lee, 2016). Whether the nucleolus could also sequester particular genes to regulate their expression remains an open question. However, this hypothesis is supported by the recent discovery of two types of NADs in mammalian cells: type I, which present heterochromatin features; and type II, enriched in developmentally regulated genes (Vertii *et al.*, 2019). Type I and II NADs could be considered as constitutive and facultative NADs, respectively. Are developmentally regulated genes still released from the nucleolar area when expressed? If this is the case, the factors responsible of the nucleolus association of NAD genes are still unknown, although some nucleolar proteins were already found to tether centromeric regions at the nucleolar periphery (Padeken *et al.*, 2013).

In addition to rRNA transcription and ribosome biogenesis, the nucleolus should be considered as a platform tethering genomic regions enriched in repressive chromatin marks. In Arabidopsis, rRNA genes derived from NOR-bearing chromosome 4 associate with the nucleolus and are actively transcribed, while the rRNA genes derived from NOR-bearing chromosome 2 are silent and excluded from the nucleolus. However, in mutants in which both chromosomal NORs are expressed, NADs also become enriched in chromosome 2 genomic regions. These results suggest that the NOR locations on the chromosome and its expression levels seem to be an important aspect of the composition of NADs (reviewed in Picart-Picolo *et al.*, 2019; Pontvianne and Grob, 2020). Identification of the NADs in different cell types and in stress conditions should help in understanding how dynamic the NAD composition is and the importance of the nucleolus function in gene regulation, but a first analysis of NADs under heat stress did not reveal significant differences compared with the control plants (Picart-Picolo *et al.*, 2020).

## The nuclear periphery

The nuclear periphery (NP) which surrounds the nucleoplasm consists of the nuclear envelope (NE), a double membrane layer with a continuum to the endoplasmatic reticulum (ER), nuclear pore complexes (NPCs), and the membrane adjoining the NPCs. The NP ensures the regulated transfer of mRNAs from the nucleus to the cytoplasm and of proteins in the reciprocal direction, and has essential roles in gene regulation and genome organization in providing attachment sites for genomic regions and chromatin. While the cell biology of the NP and the associated chromatin has been extensively reviewed (e.g. Meier *et al.*, 2017), we will here only highlight recent advances and particularly address the role of the NP in gene regulation.

The inner nuclear membrane can be associated with a protein mesh, the nuclear lamina, and interacts with transmembrane components that are part of the linker of nucleoskeleton and cytoskeleton (LINC) complex spanning the NE. The nuclear lamina in animals consists of lamins, type-5 intermediate filament proteins, and lamin-associated proteins. While some of the LINC complex members are conserved in plants, strictly defined lamins and lamin-associated genes are not present in plant genomes (Poulet *et al.*, 2017*b*). However, putative lamin analogues were initially identified in *Daucus carota* as the nuclear matrix constituent protein (NMCP) family (Masuda *et al.*, 1997). Subsequent analysis uncovered orthologues in various plant species, including Arabidopsis whose genome contains four orthologues of *DcNMCP1*, the *CROWDED NUCLEI* (*CRWN1–CRWN4*), and maize with two orthologues called *NMCP/CRWN homolog* (*NCH1* and *2*) genes (Dittmer *et al.*, 2007; Sakamoto and Takagi, 2013; Gumber *et al.*, 2019). Further lamina components were revealed by proteomic analysis of the nuclear lamina, which also identified CRWN1 and CRWN4 (Sakamoto and Takagi, 2013). Another plant-specific component of the lamina, lacking a transmembrane domain, is *KAKU4*, which interacts with CRWN1 and CRWN4 and is only present in angiosperm genomes (Goto *et al.*, 2014; Poulet *et al.*, 2017*b*). Importantly, lack of functional *CRWN1* and *KAKU4* (and other components of the NE and NP) in Arabidopsis results in nuclear morphology changes such as reduced nuclear size and increased circularity, but does not lead to obvious growth defects (Dittmer *et al.*, 2007; Sakamoto and Takagi, 2013; Wang *et al.*, 2013; Goto *et al.*, 2014). Only higher order *crwn* mutant combinations with *crwn1* result in severe dwarfism and cell death or, when all four *CRWN* genes are lacking, in lethality (Wang *et al.*, 2013). In particular, the cell death phenotype is to a large extent explained by an ectopic expression of defence genes and induction of the phytohormone salicylic acid (SA) (Choi *et al.*, 2019). Consistently, *crwn1 crwn2* double mutants are more resistant to infection by bacterial pathogens, in an SA-dependent manner (Guo *et al.*, 2017; Choi *et al.*, 2019). CRWN proteins possibly serve as direct transcriptional repressors of immunity-related genes, as CRWN1 interacts with the transcription factor NAC WITH TRANSMEMBRANE MOTIF1-LIKE9 (NTL9) which is involved in plant immunity (Guo *et al.*, 2017). Overall, lack of functional *CRWN* genes (and a disruption of nuclear lamina components in general) results in de-regulation of many genes which are involved in stress and defence responses, but also include other Gene Ontology (GO) terms (Langen *et al.*, 2014; Guo *et al.*, 2017; Mikulski *et al.*, 2019).

By microscopy analysis, it has been long known that most of the heterochromatin-containing Arabidopsis chromocentres are associated with the NE (Fransz *et al.*, 2002; Poulet *et al.*, 2017*a*). In *crwn4* mutants, chromocentres are dispersed, while the number of chromocentres is reduced in *crwn1 crwn2* double mutants (Dittmer *et al.*, 2007; Sakamoto and Takagi, 2013; Wang *et al.*, 2013). Hi-C analysis of *crwn1* and *crwn4* revealed reduced spatial separation of different chromatin compartments and therefore increased chromatin interactions between different chromatin compartments and interchromosomal interactions compared with wild-type nuclei. This indicates a reduced organization of chromatin in the *crwn1* and *crwn4* mutants and possibly higher interchromosomal compaction which may be partially explained by the decreased nuclear size (Grob *et al.*, 2014; Hu *et al.*, 2019). However, there is no overall misexpression of transposable or repetitive elements detected in any of the *crwn* single or multiple mutants (Wang *et al.*, 2013; Choi *et al.*, 2019). While the genes that are ectopically expressed in *crwn* mutants do not carry marks of constitutive heterochromatin, they are enriched in a specific chromatin state which harbours marks both of facultative heterochromatin (the PcG mark H3K27me3) and of active chromatin (H2A.Z and H3K4me3) (Sequeira-Mendes *et al.*, 2014; Choi *et al.*, 2019). This bivalent state may poise genes for fast activation upon differentiation signals or stress exposure. Indeed, two recent studies identified the genomic regions associated with the nuclear lamina, based on the association of DNA with NUCLEOPORIN1/136 and CRWN1 (Bi *et al.*, 2017; Hu *et al.*, 2019). These plant lamina-associated domains (PLADs) were enriched with repressive chromatin marks, such as H3K9me1/H3K27me1 for constitutive heterochromatin and H3K27me3 for facultative heterochromatin, and contained low expressed genes and non-accessible chromatin, suggesting that PLADs are mainly transcriptionally silent regions (Fig. 2) (reviewed in Pontvianne and Grob, 2020).

A link between PcG and the nuclear lamina may be mediated by PWO1, which interacts with both PRC2 components and CRWN1 (Hohenstatt *et al.*, 2018; Mikulski *et al.*, 2019). Mutations in *PWO1* lead to a reduction in nuclear size and misexpression of a set of genes which are also misregulated in *crwn1 crwn2* mutants (Mikulski *et al.*, 2019). Thus, PWO1 may tether PRC2 to certain genomic regions at the NP by interacting with CRWN1 or recruit specific PRC2 target genes to the NE. A direct link between chromatin/genomic regions and the NP may also be provided by the DNA-binding factor AtbZIP18 which interacts with the NE-associated protein1 (NEAP1) which is part of the LINC complex (Pawar *et al.*, 2016).

The NPC has also been extensively studied in plants, including a detailed proteomic analysis of the NPC, revealing conserved and plant-specific factors (Tamura *et al.*, 2010). NPC members have various roles in development and disease, and have been extensively reviewed (see, for example, Meier *et al.*, 2017). However, direct links to chromatin regulation have largely not been identified, except for NUCLEOPORIN1/136 (NUP1/136) which shows similar chromatin contacts to CRWN1 (Bi *et al.*, 2017; Hu *et al.*, 2019). NUP1/136 is a likely functional analogue of metazoan Nup153 which is part of the NPC basket and mediates interactions with the lamina (Smythe *et al.*, 2000). Similar to plant lamina mutants, loss of functional NUP1/136 results in smaller nuclei (Tamura and Hara-Nishimura, 2011). Further analysis is required to disentangle roles of NPC components in chromatin regulation or protein/mRNA shuffling between the nucleoplasm and the cytoplasm.

Despite recent advances, we still need to assess the dynamic association of genomic regions with the NP in response to developmental and environmental cues and reveal the functional role of the NP in gene regulation. A first analysis uncovered that light triggers a rapid repositioning of several Arabidopsis light-regulated genes from the nuclear interior to the NP when they were transcriptionally activated (Feng *et al.*, 2014). Whether this a general feature of induced genes and whether repositioning is functionally relevant needs to be determined.

## Telomeres

Telomeres are repetitive elements assembled into nucleoprotein protective caps located at the ends of linear chromosomes. They safeguard chromosomal ends against cellular exonucleases and gross chromosomal reorganization arising from the action of DNA repair machineries (Weaver, 1998; Nakamura *et al.*, 2002). While telomeres do not fit the typical definition of an NB *per se*, they are discussed here given their role in 3D chromosome organization in plants and regulation of gene expression (Fig. 1).

In plants with large genomes such as wheat, rye, or barley, interphase telomeres are polarized on one side of the nucleus in Rabl configuration (reviewed in Santos *et al.*, 2015). In some other species, such as Arabidopsis and sorghum, telomeres cluster around the nucleolus (Dong *et al.*, 1998; Fransz *et al.*, 2002) as a part of NADs (Pontvianne *et al.*, 2016*b*), introduced above. Specific telomere conformation is established during meiosis, when a telomere bouquet is seen (Bass *et al.*, 2000; Colas *et al.*, 2008). Interestingly, the observation of bouquet formation belongs to one of the earliest descriptions (Digby, 1919), even before telomeres themselves were discovered by Herman Muller and Barbara McClintock in the 1930s. They noticed an ambiguous behaviour of the terminally located DNA in irradiated cells and described the chromosome healing phenomenon (McClintock, 1941; Melek and Shippen, 1996). Later, the end replication problem was uncovered (Olovnikov, 1971), revealing that telomere erosion occurs in every S phase and, if not counteracted, limits the cellular life span (Hayflick, 1982). That further confirmed that chromosomal ends are essential functional elements and led to the most recent definition of telomeres as difficult to replicate sequences and fragile sites (reviewed in Ozer and Hickson, 2018).

Although telomeres were first discovered in plants nearly a hundred years ago, what guides their clustering, chromatin organization, or protein composition is still not clear. It is partially because plant telomeres are quite heterogeneous, thus the general model cannot be easily pictured. Their sizes range from the very short telomeres (~3 kb) in Arabidopsis to 200 kb in tobacco (Kovarik *et al.*, 1996), Moreover, plants show phylogenetic divergence at the sequence level, as the common plant telomere motif (TTTAGGG)*n* (Richards and Ausubel, 1988) is not present in all plant species (Moyzis *et al.*, 1988).

Telomerase is the most important complex interacting with telomeres, but a large number of other factors modulate telomere homeostasis. Telomere components are the dsDNA-binding factors TELOMERE REPEAT BINDING (TRB) proteins, members of the Single-Myb-Histone protein family (Marian *et al.*, 2003; Kuchar and Fajkus, 2004). The TRB proteins form speckles preferentially in the nucleolus (Dvorackova *et al.*, 2010;Schrumpfová *et al.*, 2014; Dreissig *et al.*, 2017) where they interact with factors important for telomerase biogenesis (Schorova *et al.*, 2019). The functions of the AtTRB proteins, however, are not exclusively telomeric, suggesting a link to the genome-wide chromatin remodelling via the PRC2 complex (Zhou *et al.*, 2016, 2018). Therefore, it is possible that one function of AtTRB on telomeres is to recruit other chromatin factors to incorporate H3K27me3.

The telomeric epigenetic pattern depends neither on telomere sequence composition nor on telomere lengths, and shows large variability (Adamusova *et al.*, 2020). In the case of Arabidopsis, telomeric histones are predominantly labelled by H3K9me2 and H3K27me1, with lower, but detectable enrichment of H3K4me2/me3 and H3K27me3 (Vrbsky *et al.*, 2010; Majerova *et al.*, 2014; Adamusova *et al.*, 2020). Due to the retention of H3K27me1 and H3K4me3, the Arabidopsis telomeric chromatin state is considered intermediate or bivalent (Sequeira-Mendes *et al.*, 2014; Liu *et al.*, 2016). Whether H3K27me1 and H3K4me3 co-exist at the neighbouring nucleosomes as described for other regions with bivalent chromatin (Sequeira-Mendes *et al.*, 2014) is not yet clear. Arabidopsis telomeres are also enriched with the H3.3 histone variant (Vaquero-Sedas *et al.*, 2012), which could stand in support of the view that its telomeres are not fully heterochromatic (Vaquero-Sedas and Vega-Palas, 2013). Consistently, the so-called telomere position effect (TPE), mediating silencing of telomere-adjacent genes, was not detected in Arabidopsis (Gottschling *et al.*, 1990; Aparicio *et al.*, 1991; Vrbsky *et al.*, 2010). New insights into the effects of telomeres on gene regulation could be found by using the Hi-C approach. For instance, the existence of a long-distance TPE (~10 Mb) was shown in mammals where it occurs naturally and affects the global gene expression in a telomere length-dependent manner (Kim *et al.*, 2016; Kim and Shay, 2018). Telomeres can also modulate gene transcription via long non-coding RNAs called ‘TElomeric Repeat-containing RNAs (TERRAs) (Azzalin *et al.*, 2007; Schoeftner and Blasco, 2008; Luke and Lingner, 2009; Chu *et al.*, 2017). In mammals, TERRA helps to establish telomeric heterochromatin via binding to shelterin components (Deng *et al.*, 2009), by recruitment of PRC2 (Montero *et al.*, 2018), or by competing for binding sites with the histone chaperone ALPHA THALASSEMIA-MENTAL RETARDATION X-LINKED (ATRX) not only at telomeres, but genome wide (Chu *et al.*, 2017). ATRX is known to deposit a transcription-coupled histone variant H3.3 to heterochromatic sites as well as telomeres, where the function of H3.3 is not fully understood (McKittrick *et al.*, 2004; Lewis *et al.*, 2010).

The impact of plant telomeres on long-distance interactions, the telomere gene silencing in other plant species, or participation of telomeres in formation of NBs also represent open questions.

Progressive approaches including CRISPR-based telomere labelling (Dreissig *et al.*, 2017), single RNA detection (Duncan *et al.*, 2016), Hi-C, or super-resolution microscopy have a great potential to provide new insight into the these open questions in plant telomere biology and help to understand how telomeres and telomerase modulate the chromatin in 3D nuclear space.

## Topologically associated domains

The term ‘topologically associated domains’ (TADs) describes chromatin regions that appear as ‘self-organized’ structures, formation of which is associated with chromatin compartmentalization, chromatin looping, and chromatin insulation (reviewed in Pontvianne and Grob, 2020). The invention of the Hi-C technique and its derivatives enabled researchers to investigate genome-wide chromatin organization patterns with resolution as high as 1 kb (Lieberman-Aiden *et al.*, 2009). As the most important finding made with Hi-C, TADs were originally reported in mammalian cells (Dixon *et al.*, 2012) and since then different Hi-C maps have been created, which are essentially a numeric matrix describing relative chromatin interaction frequencies between any two genomic regions. Upon visualizing a Hi-C map with colours with different intensities that correspond to chromatin interaction values, one can observe TADs as distinct squares lined up along the diagonal, in which each TAD labels a genomic region showing stronger *cis* contacts inside this TAD than interaction across its boundaries. It should be noted that nowadays TAD calling is still a challenging task on a genomic scale; rather variable results can be obtained with different algorithms.

In a 3D prospective, a TAD on a 2D Hi-C map can be considered as a self-organized chromatin domain that is relatively insulated from its neighbouring chromatin regions (Fig. 2). Such an interpretation is supported by high-resolution microscopic studies demonstrating spatial isolation of TADs (Bintu *et al.*, 2018; Szabo *et al.*, 2018; Mateo *et al.*, 2019), although these investigated TADs were only a small fraction of those identified across the genome. In animals, TAD formation has been shown to be mainly contributed by chromatin insulators [e.g. protein complexes containing CCCTC-binding factor (CTCF) and cohesin], as well as chromatin states that reflect local epigenetic marks and transcriptional activities (reviewed recently by Szabo *et al.*, 2019; Zheng and Xie, 2019; Pontvianne and Grob, 2020). Therefore, how TADs are demarcated is dependent on both the genome sequence and chromatin activities/marks, where the latter can vary from one cell type to another. The space constraint of chromatin contact patterns in TADs creates chromatin interaction specificity; in some cases, the demarcation of TADs appears to be critical for gene expression regulation and cell differentiation, such as limb development (Andrey *et al.*, 2013; Lupianez *et al.*, 2015) and oncogene activation (Flavahan *et al.*, 2016; Hnisz *et al.*, 2016; Dixon *et al.*, 2018). However, it is not always true that TADs play dominant roles in determining gene expression. For instance, a recent study on several structural variations in the *Drosophila* genome causing changes in chromatin topology (including TADs) showed that they are not predictive of changes in gene expression. These results suggest that the expression of a gene can endure alternative TAD patterns as long as it maintains the same enhancer–promoter contact profile (Ghavi-Helm *et al.*, 2019).

Over the past few years, Hi-C analyses have been conducted on many plant species showing a diversity of TAD patterns in distinct plant genomes (reviewed in Dogan and Liu, 2018; Sotelo-Silveira *et al.*, 2018; Pontvianne and Grob, 2020). First, unlike animals, not all plant species display extensive TAD patterns throughout their genomes. In particular, two closely related species in the *Brassicaceae* family do not even have TADs at their chromosome arms. Secondly, for those plants showing TAD patterns, none of them has TADs featured with the presence of chromatin loops that connect TAD borders, which is a prominent characteristic of many animal TADs. Thirdly, distinct from animals TADs, plant TADs do not show pattern conservation between syntenic regions in different species, and genes residing in the same plant TAD lack co-expression (Dong *et al.*, 2017; Zhou *et al.*, 2018). Nevertheless, similar gene transcription and epigenetic profiles at plant and animal TAD borders indicate that it is a feature of these studied eukaryotes to have active and open chromatin preferentially associated with these chromatin regions (reviewed in Dogan and Liu, 2018; Sotelo-Silveira *et al.*, 2018; Pontvianne and Grob, 2020). This notion is further supported by comparative Hi-C studies. For instance, in a recent study comparing TAD patterns among different plant tissues, Dong *et al*. (2019) showed that tissue-specific TAD borders in rice and maize often overlap with gene up-regulation. Another example comes from comparisons of TAD patterns in diploid and subgenomes of tetraploid (formed via polyploidization) cotton genomes, which shows that conserved TAD boundary regions tend to have a higher level of chromatin accessibility and the euchromatin histone mark H3K4me3 than do non-conserved regions (Wang *et al.*, 2018). Accordingly, this study reveals that non-conserved TAD borders have a higher probability of displaying differential gene expression. At present, studies comparing TAD structures in normally growing plants and those in plants responding to biotic and/or abiotic stimuli are extremely limited, and the extent to which TAD formation regulates plant growth and development is unclear.

Mechanisms underlying plant TAD formation are unknown at the moment. CTCF-mediated TAD formation in mammals happens along with the formation of chromatin loops that link TAD boundary regions (Sanborn *et al.*, 2015; Fudenberg *et al.*, 2016). This mechanism, which results in strong chromatin insulation at TAD borders, seems to be missing in plant genomes, as plants do not have *CTCF*-like genes. However, the strong correlation between active local gene expression and TAD borders suggests that plant TADs are shaped by transcriptional regulation. Recent motif analyses of rice TAD border regions revealed enrichment of sequences recognized by TCP (TEOSINTE BRANCHED 1, CYCLOIDEA, PCF1) and bZIP (basic leucine zipper) proteins, which belong to two large plant transcription factor families (Dogan and Liu, 2018). Further investigation of their potential roles in chromatin insulation would be helpful for plant scientists to better understand plant TAD formation. Additional potential plant TAD formation mechanisms have been discussed recently. Based on surveying TAD patterns from different plants, Stam *et al.* (2019) hypothesized that TAD formation, as well as TAD identification, are feasible in plant genomes bearing large-sized and dispersed repeat-containing chromatin regions. In other words, plant TADs largely reflect the spatial separation of repressive chromatin domains from their flanking chromatin. This idea can be tested by checking chromatin interaction patterns of TAD-containing genomic regions after inserting them into the Arabidopsis genome. Technically, the transformation-competent artificial chromosome vector (TAC) system enables the insertion of large genomic DNA (50–100 kb) into host plants (Liu *et al.*, 1999, 2002), which is sufficient to harbour most TADs identified in the rice genome (Liu *et al.*, 2017). In summary, the very limited knowledge of plant TAD function and formation calls for more efforts to be made to better understand 3D plant genomes.

## Nuclear subdomains are formed by intrinsically disordered domain proteins via liquid–liquid phase separation

Considering the information discussed in the previous sections, the question arises of how the nuclear domains are assembled without the formation of a surrounding membrane, which is necessary in organelles to maintain a local concentration of cellular components and to separate different metabolic activities. The answer lies in the liquid-like behaviour of biomolecular structures that undergo phase transition. The basic principle is that above a critical concentration, proteins aggregate and form a network of interactions with other molecules, resulting in dense liquid droplets. The first evidence of such a mechanism came from studies of segregating RNA and protein-rich P granules in *Caenorhabditis elegans* (Brangwynne *et al.*, 2009). Their posterior localization in the nematode embryo involves condensation of its macromolecular components. During the past few years, an increasing number of studies in different organisms have provided evidence that the physicochemical forces underlie the segregation of biological macromolecules into droplets, leading to the assembly of membraneless compartments in animals (Li *et al.*, 2012; Feric *et al.*, 2016; Langdon and Gladfelter, 2018; Maharana *et al.*, 2018; Sabari *et al.*, 2018; Shin *et al.*, 2018; Boeynaems *et al.*, 2019; Falk *et al.*, 2019; [Bibr CIT0261][Bibr CIT0271]) and in plants (Fang *et al.*, 2019; Zhang *et al.*, 2019; Zhou *et al.*, 2019).

Liquid–liquid phase separation (LLPS) or liquid phase condensation is the de-mixing of a homogeneous solution of proteins into two liquid phases where one is enriched for the protein (reviewed in Alberti, 2017; Strom and Brangwynne, 2019). Multiple droplets with similar content can form a compartment. The value of the critical concentration at which phase transition occurs depends on several factors, including intrinsic molecular properties of the protein domains and the nature and intensity of the interaction between the macromolecules. For example, a high RNA–protein ratio and a high number of interacting molecular domains (multivalency) reduce the critical concentration and hence induce LLPS (Li *et al.*, 2012; Maharana *et al.*, 2018). In this context, post-translational modifications, such as phosphorylation or methylation, which can alter protein interactions, play an important role in phase separation, as is further discussed.

Several chromatin proteins have so-called intrinsically disordered regions (IDRs) providing the biomolecules with an intrinsic capacity for LLPS required to form nuclear bodies involving chromatin. The extensively studied non-histone chromatin protein family, Heterochromatin Protein 1 (HP1), first described in *Drosophila* (James and Elgin, 1986), is a prominent component of heterochromatin. The human HP1α can phase separate and induce compaction of associated DNA (Larson *et al.*, 2017). The formation of phase-separated droplets was promoted by phosphorylation of HP1α and by DNA binding, confirming the importance of post-translational modifications in LLPS. De-mixing into droplets has also been demonstrated for the *Drosophila* HP1a protein and forms the driving force behind the assemblage of heterochromatin domains (Strom *et al.*, 2017). Arabidopsis contains a functional equivalent of HP1, agenet domain-containing protein (ADCP), which binds to methylated H3K9 and localizes to chromocentres. ADCP can also undergo liquid–liquid de-mixing and forms DNA-rich droplets upon methylated H3K9 recognition (Zhang *et al.*, 2018; Zhao *et al.*, 2019). These data provide evidence that animal HP1 and plant ADCP mediate the formation of constitutive heterochromatic chromocentres via phase separation.

The formation of PcBs in mammalian nuclei is established by Chromobox 2 (CBX2) via LLPS. CBX2 is the homologue of chromodomain-containing Polycomb (Pc) protein of *Drosophila* and a component of PRC1. CBX2 undergoes phase separation, thereby compacting PcG-associated chromatin (Tatavosian *et al.*, 2019). Phosphorylation of serines in CBX2 is an important step in the phase separation event. The low-complexity disordered region in CBX2 appears important for both LLPS and chromatin compaction, clearly indicating the link between the two processes (Plys *et al.*, 2019). Although plant LHP1 has the chromodomain and the chromoshadow domain (like HP1), it does not bind to methylated H3K9. Instead, LHP1 binds to H3K27me3 and maintains repression of PRC2 target genes (Turck *et al.*, 2007; Zhang *et al.*, 2007; Exner *et al.*, 2009), being considered the plant equivalent of Pc/CBX2 in PRC1. In addition, and as we have previously commented, LHP1 forms nuclear speckles (Libault *et al.*, 2005). Both nuclear distribution in speckles and H3K27me3 interaction are dependent on the presence of the chromodomain (Exner *et al.*, 2009). Whether LHP1 is involved in LLPS activity and responsible for PcB formation is very likely but needs to be determined. In this context, VERNALIZATION 1 (VRN1) might be an interesting factor. The plant-specific VRN1 mediates vernalization and binds DNA via its two B3 domains in a sequence-non-specific manner to repress gene targets such as *FLC* (Levy *et al.*, 2002). VRN1 is considered a member of PRC1 in plants although it is not a core component (Y. Huang *et al.*, 2019; reviewed in Holec and Berger, 2012; Kim and Sung, 2014). VRN1 has been demonstrated to undergo LLPS when it interacts with DNA giving rise to nuclear speckles. Both B3 domains and the IDR located in between them are essential for the phase separation event (Zhou *et al.*, 2019).

Another protein family involved in heterochromatin-linked LLPS are MORC ATPases that catalyse changes in chromatin structure in plants and animals (Koch *et al.*, 2017). In mammals, MORC proteins dimerize via their ATPase domain, which enables MORC to associate with chromatin and form NBs via LLPS (Zhang *et al.*, 2019. Its CW-type zinc finger domain can inhibit binding to DNA, forming an inactive state of MORC. The inactive state is released when CW interacts with H3K4me3 (Zhang, 2019). In Arabidopsis, AtMORCs are concentrated in discrete NBs at the boundary of chromocentres, while AtMORC4 and AtMORC7 also appear more diffuse in the nucleoplasm (Harris *et al.*, 2016). However, AtMORCs do not have a CW domain (Langen *et al.*, 2014), which makes it unclear how they are directed to chromatin.

The nucleolus, the most prominent NB (see above), is a clear example of a nuclear compartment formed by LLPS. Fibrillarin (FIB1) in the dense fibrillar component and nucleophosmin (NPM1) in the granular component of the nucleolus can phase separate *in vitro* into droplets as the nucleolar compartments do (Feric *et al.*, 2016; Yao *et al.*, 2019). In addition, both NPM1 and FIB1 require the presence of rRNA to phase separate into droplets, indicating the importance of protein–RNA interactions in compartment formation. The disordered regions in FIB1 and NPM1 are necessary for the phase separation process, while the RNA-interacting domains are needed to segregate into the proper nucleolar subcompartment (Yao *et al.*, 2019). Moreover, the latter promotes the sorting of correctly folded but not unfolded pre-rRNA into the DFC, pointing at a delicate interaction between multivalent FIB1 and rDNA transcripts. The tripartite organization of the nucleolus can be disturbed when aberrant, intrinsically disordered proteins interfere with the nucleolar phase separation. For example, when aberrant arginine-enriched dipeptide domains of the C9Orf72 protein interact with NPM1, the GC droplets dissolve. This phenomenon is observed in the human disease amyotrophic lateral sclerosis (ALS) (White *et al.*, 2019).

The list of studies on LLPS-mediated formation of NBs is still growing, indicating that phase transitions are key mechanisms for nuclear organization. For example, phosphatidylinositols, in particular phosphorylated PIP2, can play an important role in nuclear compartment formation since they have been detected in the nucleoplasm, nuclear speckles, and nucleolus. They interact with >300 nuclear proteins (Lewis *et al.*, 2011), including RNA polymerase II, RNA polymerase I, and FIB1, and associate with NORs during mitosis in human cells (Yildirim *et al.*, 2013; Sobol *et al.*, 2018). They form 40–100 nm sized nuclear lipid islets (NLIs), which have been observed in many organisms, including animals, plants, and yeast (Sztacho *et al.*, 2019). A direct interaction between phosphatidylinositols and chromatin is found for phosphatidyl 5-phosphate, which facilitates the binding of UHRF1, a ubiquitin-like PHD and RING finger domain protein, to methylated histone H3K9, thereby regulating epigenetic states (Gelato *et al.*, 2014).

Considering that the histone code is based on the post-translational modification of histone tails, it is likely that histone modifications play an important role in the organization of chromatin via LLPS. Indeed, in a phase separation experiment with reconstituted chromatin and physiological salt, nucleosomes with histone tails clearly showed LLPS, whereas ‘tail-less’ nucleosomes did not (Gibson *et al.*, 2019). Moreover, linker histone H1 and nucleosome linker length promote phase separation, while histone acetylation antagonizes chromatin phase separation. In addition, the interaction between chromodomain proteins and methylated histone H3K9 leads to condensed droplets in a tube, indicating that LLPS is a major driving force behind heterochromatin formation (Wang *et al.*, 2019).

LLPS has also been associated with the formation of euchromatin compartments. In mammalian cells, the co-activators Mediator (Med1) and Bromodomain-containing protein 4 (BRD4) are enriched at clusters of enhancers and transcription machinery components, so-called super enhancers (Pott and Lieb, 2015; Sabari *et al.*, 2018). It is proposed that Med1 and BRD4 form phase-separated condensates via their IDRs which enable compartmentalization with enhancers and transcription factors (Sabari *et al.*, 2018).

In conclusion, the LLPS studies point to a universal biological mechanism that explains the segregation of nuclear proteins into concentrated, small areas and the formation of chromosomal compartments with a specific epigenetic signature. The discovery of membraneless compartments by LLPS enables us to understand the dynamics of individual RNAs inside the nucleus (Pitchiaya *et al.*, 2019). The intrinsic molecular properties and a critical concentration of the associating macromolecules determine the unique identity of NBs, while specific epigenetic features along the chromosomes contribute to local compartment formation within chromatin. In fact, almost every topological change in the nucleoplasm is subject to LLPS forces.

## Conclusion

Technological advances in the analysis of chromatin organization, together with the application of next-generation sequencing (NGS) to decipher in more depth the genetic and epigenetic information of an increased number of plant species, have allowed the existence of several compartments in the 3D nucleus to be highlighted and are helping to uncover connections between regulatory domains of the 3D genome. Nevertheless, how spatial and structural organization of the nucleus influences its function is far from being understood. Recent discoveries in this area have indeed proven to be key in depicting the complexity of gene expression regulation within the reduced nuclear space as we have discussed. However, caution needs to be present in attributing functions to 3D nuclear organization. The intrinsic capacity of chromatin to be plastic allied to adjustments of chromatin organization and nuclear domain arrangements in relation to plant species, specific transcriptional requirements, developmental stages, cell and tissue type, and epigenetic and stress factors can aid in allowing fast changes in gene expression and thus to induce quicker responses to specific requirements (Fig. 1).

LLPS is increasingly emerging as an important player in 3D genomics. Understanding the formation and organization of highly conserved and plant-specific nuclear domains in response to external and internal cues will contribute to our understanding of the regulation of these processes and plant phenotypes but also, most probably, to the discovery of future molecular tools. On the other hand, this complex nuclear dynamism illustrates well the difficulty in finding universal rules for making direct connections between 3D nuclear organization and predictable gene expression. Furthermore, chromatin-mediated regulation of genome accessibility may also be involved in molecular memories of responses given to different types of stimuli, but very little is known about perpetuating chromatin states and even less about inheritable gene expression patterns between generations as a way to ensure more prompt responses to challenges (Fig. 1) (reviewed in Mozgová *et al.*, 2019). Increasing the amount of knowledge on the connections between interphase nuclear domains and the regulation of gene expression may enable identification of novel chromatin-based markers, ideally stable and associated with predictable impacts on plant traits, which in future plant breeding programmes can aid in improving crop growth under suboptimal environmental conditions.

## Abbreviations:

CB: Cajal body or coiled body
CENH3: centromere-specific histone H3 variant
CRWN: CROWDED NUCLEI
FISH: fluorescence *in situ* hybridization
GISH: genomic *in situ* hybridization
HP1: Heterochromatin Protein 1
IDR: intrinsically disordered region
LAD: lamina-associated domain
LLPS: liquid–liquid phase separation
NAD: nucleolar-associated domain
NB: nuclear body
NE: muclear envelope
NMCP: nuclear matrix constituent protein
NOR: nucleolus organizing region
NP: nuclear periphery
NPC: nuclear pore complex
NUP1/136: NUCLEOPORIN1/136
PB: photobody
Pc: Polycomb
PcB: Polycomb body
PcG: Polycomb-group
PRC: Polycomb Repressive Complex
PLAD: plant lamina-associated domain
PWO1: DOMAIN INTERACTOR OF POLYCOMBS1
TAD: topologically associated domain
TrF: transcription factory
SpS: splicing speckle
TERRA: TElomeric Repeat-containing RNA
TRB: TELOMERE REPEAT BINDING
VRN1: VERNALIZATION 1

## References

[CIT0001] Adamusova K , Khosravi S, Fujimoto S, Matsunaga S, Fajkus J, Fojtová M. 2020. Two combinatorial patterns of telomere histone marks in plants with canonical and non-canonical telomere repeats. The Plant Journal 102, 678–687. 31834959 10.1111/tpj.14653

[CIT0002] Adriaens C , Serebryannyy LA, Feric M, et al. 2018. Blank spots on the map: some current questions on nuclear organization and genome architecture. Histochemistry and Cell Biology 150, 579–592. 30238154 10.1007/s00418-018-1726-1 PMC6290477

[CIT0003] Albert PS , Zhang T, Semrau K, Rouillard JM, Kao YH, Wang CR, Danilova TV, Jiang J, Birchler JA. 2019. Whole-chromosome paints in maize reveal rearrangements, nuclear domains, and chromosomal relationships. Proceedings of the National Academy of Sciences, USA 116, 1679–1685. 10.1073/pnas.1813957116 PMC6358699 30655344

[CIT0004] Alberti S . 2017. Phase separation in biology. Current Biology 27, R1097–R1102. 29065286 10.1016/j.cub.2017.08.069

[CIT0005] Andrey G , Montavon T, Mascrez B, Gonzalez F, Noordermeer D, Leleu M, Trono D, Spitz F, Duboule D. 2013. A switch between topological domains underlies HoxD genes collinearity in mouse limbs. Science 340, 1234167. 23744951 10.1126/science.1234167

[CIT0006] Anton T , Bultmann S, Leonhardt H, Markaki Y. 2014. Visualization of specific DNA sequences in living mouse embryonic stem cells with a programmable fluorescent CRISPR/Cas system. Nucleus 5, 163–172. 24637835 10.4161/nucl.28488 PMC4049922

[CIT0007] Aparicio OM , Billington BL, Gottschling DE. 1991. Modifiers of position effect are shared between telomeric and silent mating-type loci in *S. cerevisiae*. Cell 66, 1279–1287. 1913809 10.1016/0092-8674(91)90049-5

[CIT0008] Aragon-Alcaide L , Reader S, Beven A, Shaw P, Miller T, Moore G. 1997. Association of homologous chromosomes during floral development. Current Biology 7, 905–908. 9382806 10.1016/s0960-9822(06)00383-6

[CIT0009] Audas TE , Jacob MD, Lee S. 2012. Immobilization of proteins in the nucleolus by ribosomal intergenic spacer noncoding RNA. Molecular Cell 45, 147–157. 22284675 10.1016/j.molcel.2011.12.012

[CIT0010] Audas TE , Lee S. 2016. Stressing out over long noncoding RNA. Biochimica et Biophysica Acta 1859, 184–191. 26142536 10.1016/j.bbagrm.2015.06.010 PMC9479161

[CIT0011] Azzalin CM , Reichenbach P, Khoriauli L, Giulotto E, Lingner J. 2007. Telomeric repeat containing RNA and RNA surveillance factors at mammalian chromosome ends. Science 318, 798–801. 17916692 10.1126/science.1147182

[CIT0012] Baroux C , Pecinka A, Fuchs J, Kreth G, Schubert I, Grossniklaus U. 2017. Non-random chromosome arrangement in triploid endosperm nuclei. Chromosoma 126, 115–124. 26892012 10.1007/s00412-016-0578-5

[CIT0013] Bass HW , Riera-LizarazuO, AnanievEV, BordoliSJ, RinesHW, PhillipsRL, SedatJW, AgardDA, CandeWZ. 2000. Evidence for the coincident initiation of homolog pairing and synapsis during the telomere-clustering (bouquet) stage of meiotic prophase. Journal of Cell Science113, 1033–1042.1068315110.1242/jcs.113.6.1033

[CIT0014] Bassett CL . 2012. Cajal bodies and plant RNA metabolism. Critical Reviews in Plant Sciences 31, 258–270.

[CIT0015] Baudement MO , Cournac A, Court F, et al. 2018. High-salt-recovered sequences are associated with the active chromosomal compartment and with large ribonucleoprotein complexes including nuclear bodies. Genome Research 28, 1733–1746. 30287550 10.1101/gr.237073.118 PMC6211644

[CIT0016] Bauer D , Viczián A, Kircher S, et al. 2004. Constitutive photomorphogenesis 1 and multiple photoreceptors control degradation of phytochrome interacting factor 3, a transcription factor required for light signaling in Arabidopsis. The Plant Cell 16, 1433–1445. 15155879 10.1105/tpc.021568 PMC490037

[CIT0017] Benoit M , Simon L, Desset S, Duc C, Cotterell S, Poulet A, Le Goff S, Tatout C, Probst AV. 2019. Replication-coupled histone H3.1 deposition determines nucleosome composition and heterochromatin dynamics during Arabidopsis seedling development. New Phytologist 221, 385–398. 10.1111/nph.15248 29897636

[CIT0018] Bi X , Cheng YJ, Hu B, Ma X, Wu R, Wang JW, Liu C. 2017. Nonrandom domain organization of the Arabidopsis genome at the nuclear periphery. Genome Research 27, 1162–1173. 28385710 10.1101/gr.215186.116 PMC5495068

[CIT0019] Bintu B , Mateo LJ, Su JH, Sinnott-Armstrong NA, Parker M, Kinrot S, Yamaya K, Boettiger AN, Zhuang X. 2018. Super-resolution chromatin tracing reveals domains and cooperative interactions in single cells. Science 362, eaau1783. 30361340 10.1126/science.aau1783 PMC6535145

[CIT0020] Boeynaems S , Holehouse AS, Weinhardt V, et al. 2019. Spontaneous driving forces give rise to protein–RNA condensates with coexisting phases and complex material properties. Proceedings of the National Academy of Sciences, USA 116, 7889–7898. 10.1073/pnas.1821038116 PMC6475405 30926670

[CIT0021] Boisvert FM , van Koningsbruggen S, Navascués J, Lamond AI. 2007. The multifunctional nucleolus. Nature Reviews. Molecular Cell Biology 8, 574–585. 17519961 10.1038/nrm2184

[CIT0022] Boudonck K , Dolan L, Shaw PJ. 1998. Coiled body numbers in the Arabidopsis root epidermis are regulated by cell type, developmental stage and cell cycle parameters. Journal of Cell Science 111, 3687–3694. 9819359 10.1242/jcs.111.24.3687

[CIT0023] Boudonck K , Dolan L, Shaw PJ. 1999. The movement of coiled bodies visualized in living plant cells by the green fluorescent protein. Molecular Biology of the Cell 10, 2297–2307. 10397766 10.1091/mbc.10.7.2297 PMC25444

[CIT0024] Boulon S , Westman BJ, Hutten S, Boisvert FM, Lamond AI. 2010. The nucleolus under stress. Molecular Cell 40, 216–227. 20965417 10.1016/j.molcel.2010.09.024 PMC2987465

[CIT0025] Brangwynne CP , Eckmann CR, Courson DS, Rybarska A, Hoege C, Gharakhani J, Jülicher F, Hyman AA. 2009. Germline P granules are liquid droplets that localize by controlled dissolution/condensation. Science 324, 1729–1732. 19460965 10.1126/science.1172046

[CIT0026] Brown DD , Gurdon JB. 1964. Absence of ribosomal RNA synthesis in the anucleolate mutant of Xenopus. Proceedings of the National Academy of Sciences, USA 51, 139–146. 10.1073/pnas.51.1.139 PMC300879 14106673

[CIT0027] Buckley MS , Lis JT. 2014. Imaging RNA polymerase II transcription sites in living cells. Current Opinion in Genetics & Development 25, 126–130. 24794700 10.1016/j.gde.2014.01.002 PMC5497218

[CIT0028] Calil IP , Quadros IPS, Araújo TC, et al. 2018. A WW domain-containing protein forms immune nuclear bodies against Begomoviruses. Molecular Plant 11, 1449–1465. 30296599 10.1016/j.molp.2018.09.009

[CIT0029] Calonje M , Sanchez R, Chen L, Sung ZR. 2008. EMBRYONIC FLOWER1 participates in polycomb group-mediated AG gene silencing in Arabidopsis. The Plant Cell 20, 277–291. 18281509 10.1105/tpc.106.049957 PMC2276442

[CIT0030] Carpentier MC , Picart-Picolo A, Pontvianne F. 2018. A method to identify nucleolus-associated chromatin domains (NADs). Methods in Molecular Biology 1675, 99–109. 29052188 10.1007/978-1-4939-7318-7_7

[CIT0031] Chandrasekhara C , Mohannath G, Blevins T, Pontvianne F, Pikaard CS. 2016. Chromosome-specific NOR inactivation explains selective rRNA gene silencing and dosage control in Arabidopsis. Genes & Development 30, 177–190. 26744421 10.1101/gad.273755.115 PMC4719308

[CIT0032] Chen M , Chory J. 2011. Phytochrome signaling mechanisms and the control of plant development. Trends in Cell Biology 21, 664–671. 21852137 10.1016/j.tcb.2011.07.002 PMC3205231

[CIT0033] Cheutin T , Cavalli G. 2014. Polycomb silencing: from linear chromatin domains to 3D chromosome folding. Current Opinion in Genetics & Development 25, 30–37. 24434548 10.1016/j.gde.2013.11.016

[CIT0034] Choi J , Strickler SR, Richards EJ. 2019. Loss of CRWN nuclear proteins induces cell death and salicylic acid defense signaling. Plant Physiology 179, 1315–1329. 30696746 10.1104/pp.18.01020 PMC6446779

[CIT0035] Chu HP , Cifuentes-Rojas C, Kesner B, Aeby E, Lee HG, Wei C, Oh HJ, Boukhali M, Haas W, Lee JT. 2017. TERRA RNA antagonizes ATRX and protects telomeres. Cell 170, 86–101.e16. 28666128 10.1016/j.cell.2017.06.017 PMC5552367

[CIT0036] Ciabrelli F , Cavalli G. 2015. Chromatin-driven behavior of topologically associating domains. Journal of Molecular Biology 427, 608–625. 25280896 10.1016/j.jmb.2014.09.013

[CIT0037] Colas I , Shaw P, Prieto P, Wanous M, Spielmeyer W, Mago R, Moore G. 2008. Effective chromosome pairing requires chromatin remodeling at the onset of meiosis. Proceedings of the National Academy of Sciences, USA 105, 6075–6080. 10.1073/pnas.0801521105 PMC2329686 18417451

[CIT0038] Collier S , Pendle A, Boudonck K, van Rij T, Dolan L, Shaw P. 2006. A distant coilin homologue is required for the formation of cajal bodies in Arabidopsis. Molecular Biology of the Cell 17, 2942–2951. 16624863 10.1091/mbc.E05-12-1157 PMC1483031

[CIT0039] Concia L , Veluchamy A, Ramirez-Prado JS, et al. 2020. Wheat chromatin architecture is organized in genome territories and transcription factories. Genome Biology 21, 104. 32349780 10.1186/s13059-020-01998-1 PMC7189446

[CIT0040] Cremer T , Cremer C. 2001. Chromosome territories, nuclear architecture and gene regulation in mammalian cells. Nature Reviews. Genetics 2, 292–301. 10.1038/35066075 11283701

[CIT0041] Cremer T , Cremer M. 2010. Chromosome territories. Cold Spring Harbor Perspectives in Biology 2, a003889. 20300217 10.1101/cshperspect.a003889 PMC2829961

[CIT0042] Del Prete S , Arpón J, Sakai K, Andrey P, Gaudin V. 2014. Nuclear architecture and chromatin dynamics in interphase nuclei of *Arabidopsis thaliana*. Cytogenetic and Genome Research 143, 28–50. 24992956 10.1159/000363724

[CIT0043] Del Prete S , Mikulski P, Schubert D, Gaudin V. 2015. One, two, three: polycomb proteins hit all dimensions of gene regulation. Genes 6, 520–542. 26184319 10.3390/genes6030520 PMC4584315

[CIT0044] Deng Z , Norseen J, Wiedmer A, Riethman H, Lieberman PM. 2009. TERRA RNA binding to TRF2 facilitates heterochromatin formation and ORC recruitment at telomeres. Molecular Cell 35, 403–413. 19716786 10.1016/j.molcel.2009.06.025 PMC2749977

[CIT0045] De Storme N , Keçeli BN, Zamariola L, Angenon G, Geelen D. 2016. CENH3–GFP: a visual marker for gametophytic and somatic ploidy determination in *Arabidopsis thaliana*. BMC Plant Biology 16, 1. 26728271 10.1186/s12870-015-0700-5 PMC4700667

[CIT0046] Digby L . 1919. On the archesporial and meiotic mitoses of Osmunda. Annals of Botany 2, 135–172.

[CIT0047] Dittmer TA , Stacey NJ, Sugimoto-Shirasu K, Richards EJ. 2007. LITTLE NUCLEI genes affecting nuclear morphology in *Arabidopsis thaliana*. The Plant Cell 19, 2793–2803. 17873096 10.1105/tpc.107.053231 PMC2048703

[CIT0048] Dixon JR , Selvaraj S, Yue F, Kim A, Li Y, Shen Y, Hu M, Liu JS, Ren B. 2012. Topological domains in mammalian genomes identified by analysis of chromatin interactions. Nature 485, 376–380. 22495300 10.1038/nature11082 PMC3356448

[CIT0049] Dixon JR , Xu J, Dileep V, et al. 2018. Integrative detection and analysis of structural variation in cancer genomes. Nature Genetics 50, 1388–1398. 30202056 10.1038/s41588-018-0195-8 PMC6301019

[CIT0050] Docquier S , Tillemans V, Deltour R, Motte P. 2004. Nuclear bodies and compartmentalization of pre-mRNA splicing factors in higher plants. Chromosoma 112, 255–266. 14740228 10.1007/s00412-003-0271-3

[CIT0051] Doğan ES , Liu C. 2018. Three-dimensional chromatin packing and positioning of plant genomes. Nature Plants 4, 521–529. 30061747 10.1038/s41477-018-0199-5

[CIT0052] Dolata J , Taube M, Bajczyk M, Jarmolowski A, Szweykowska-Kulinska Z, Bielewicz D. 2018. Regulation of plant microprocessor function in shaping microRNA landscape. Frontiers in Plant Science 9, 753. 29922322 10.3389/fpls.2018.00753 PMC5996484

[CIT0053] Dong F , Jiang J. 1998. Non-Rabl patterns of centromere and telomere distribution in the interphase nuclei of plant cells. Chromosome Research 6, 551–558. 9886774 10.1023/a:1009280425125

[CIT0054] Dong P , Tu X, Chu PY, Lü P, Zhu N, Grierson D, Du B, Li P, Zhong S. 2017. 3D Chromatin architecture of large plant genomes determined by local A/B compartments. Molecular Plant 10, 1497–1509. 29175436 10.1016/j.molp.2017.11.005

[CIT0055] Dong P , Tu X, Li H, Zhang J, Grierson D, Li P, Zhong S. 2020. Tissue-specific Hi-C analyses of rice, foxtail millet and maize suggest non-canonical function of plant chromatin domains. Journal of Integrative Plant Biology 62, 201–217. 30920762 10.1111/jipb.12809

[CIT0056] Dreissig S , Schiml S, Schindele P, Weiss O, Rutten T, Schubert V, Gladilin E, Mette MF, Puchta H, Houben A. 2017. Live-cell CRISPR imaging in plants reveals dynamic telomere movements. The Plant Journal 91, 565–573. 28509419 10.1111/tpj.13601 PMC5599988

[CIT0057] Duncan S , Olsson TSG, Hartley M, Dean C, Rosa S. 2016. A method for detecting single mRNA molecules in *Arabidopsis thaliana*. Plant Methods 12, 13. 28035231 10.1186/s13007-016-0114-x PMC5192599

[CIT0058] Dundr M . 2012. Nuclear bodies: multifunctional companions of the genome. Current Opinion in Cell Biology 24, 415–422. 22541757 10.1016/j.ceb.2012.03.010 PMC3372688

[CIT0059] Dvořáčková M , Fojtová M, Fajkus J. 2015. Chromatin dynamics of plant telomeres and ribosomal genes. The Plant Journal 83, 18–37. 25752316 10.1111/tpj.12822

[CIT0060] Dvorácková M , Rossignol P, Shaw PJ, Koroleva OA, Doonan JH, Fajkus J. 2010. AtTRB1, a telomeric DNA-binding protein from Arabidopsis, is concentrated in the nucleolus and shows highly dynamic association with chromatin. The Plant Journal 61, 637–649. 19947985 10.1111/j.1365-313X.2009.04094.x

[CIT0061] Exner V , Aichinger E, Shu H, Wildhaber T, Alfarano P, Caflisch A, Gruissem W, Köhler C, Hennig L. 2009. The chromodomain of LIKE HETEROCHROMATIN PROTEIN 1 is essential for H3K27me3 binding and function during Arabidopsis development. PLoS One 4, e5335. 19399177 10.1371/journal.pone.0005335 PMC2670505

[CIT0062] Fajkus J , Kovarík A, Královics R. 1996. Telomerase activity in plant cells. FEBS Letters 391, 307–309. 8764995 10.1016/0014-5793(96)00757-0

[CIT0063] Falk M , Feodorova Y, Naumova N, et al. 2019. Heterochromatin drives compartmentalization of inverted and conventional nuclei. Nature 570, 395–399. 31168090 10.1038/s41586-019-1275-3 PMC7206897

[CIT0064] Fang X , Wang L, Ishikawa R, et al. 2019. Arabidopsis FLL2 promotes liquid–liquid phase separation of polyadenylation complexes. Nature 569, 265–269. 31043738 10.1038/s41586-019-1165-8 PMC6625965

[CIT0065] Fang Y , Spector DL. 2007. Identification of nuclear dicing bodies containing proteins for microRNA biogenesis in living Arabidopsis plants. Current Biology 17, 818–823. 17442570 10.1016/j.cub.2007.04.005 PMC1950788

[CIT0066] Fei J , Jadaliha M, Harmon TS, et al. 2017. Quantitative analysis of multilayer organization of proteins and RNA in nuclear speckles at super resolution. Journal of Cell Science 130, 4180–4192. 29133588 10.1242/jcs.206854 PMC5769577

[CIT0067] Feng CM , Qiu Y, Van Buskirk EK, Yang EJ, Chen M. 2014. Light-regulated gene repositioning in Arabidopsis. Nature Communications 5, 3027. 10.1038/ncomms4027 PMC4136543 24390011

[CIT0068] Feng S , Jacobsen SE. 2011. Epigenetic modifications in plants: an evolutionary perspective. Current Opinion in Plant Biology 14, 179–186. 21233005 10.1016/j.pbi.2010.12.002 PMC3097131

[CIT0069] Feric M , Vaidya N, Harmon TS, Mitrea DM, Zhu L, Richardson TM, Kriwacki RW, Pappu RV, Brangwynne CP. 2016. Coexisting liquid phases underlie nucleolar subcompartments. Cell 165, 1686–1697. 27212236 10.1016/j.cell.2016.04.047 PMC5127388

[CIT0070] Fitzgerald MS , Shakirov EV, Hood EE, McKnight TD, Shippen DE. 2001. Different modes of de novo telomere formation by plant telomerases. The Plant Journal 26, 77–87. 11359612 10.1046/j.1365-313x.2001.01010.x

[CIT0071] Flavahan WA , Drier Y, Liau BB, Gillespie SM, Venteicher AS, Stemmer-Rachamimov AO, Suvà ML, Bernstein BE. 2016. Insulator dysfunction and oncogene activation in IDH mutant gliomas. Nature 529, 110–114. 26700815 10.1038/nature16490 PMC4831574

[CIT0072] Fransz P , De Jong JH, Lysak M, Castiglione MR, Schubert I. 2002. Interphase chromosomes in Arabidopsis are organized as well defined chromocenters from which euchromatin loops emanate. Proceedings of the National Academy of Sciences, USA 99, 14584–14589. 10.1073/pnas.212325299 PMC137926 12384572

[CIT0073] Friesner JD , Liu B, Culligan K, Britt AB. 2005. Ionizing radiation-dependent gamma-H2AX focus formation requires ataxia telangiectasia mutated and ataxia telangiectasia mutated and Rad3-related. Molecular Biology of the Cell 16, 2566–2576. 15772150 10.1091/mbc.E04-10-0890 PMC1087258

[CIT0074] Fudenberg G , Imakaev M, Lu C, Goloborodko A, Abdennur N, Mirny LA. 2016. Formation of chromosomal domains by loop extrusion. Cell Reports 15, 2038–2049. 27210764 10.1016/j.celrep.2016.04.085 PMC4889513

[CIT0075] Fujioka Y , Utsumi M, Ohba Y, Watanabe Y. 2007. Location of a possible miRNA processing site in SmD3/SmB nuclear bodies in Arabidopsis. Plant & Cell Physiology 48, 1243–1253. 17675322 10.1093/pcp/pcm099

[CIT0076] Fujimoto S , Sugano SS, Kuwata K, Osakabe K, Matsunaga S. 2016. Visualization of specific repetitive genomic sequences with fluorescent TALEs in *Arabidopsis thaliana*. Journal of Experimental Botany 67, 6101–6110. 27811079 10.1093/jxb/erw371 PMC5100022

[CIT0077] Galganski L , Urbanek MO, Krzyzosiak WJ. 2017. Nuclear speckles: molecular organization, biological function and role in disease. Nucleic Acids Research 45, 10350–10368. 28977640 10.1093/nar/gkx759 PMC5737799

[CIT0078] Gall JG . 2000. Cajal bodies: the first 100 years. Annual Review of Cell and Developmental Biology 16, 273–300. 10.1146/annurev.cellbio.16.1.273 11031238

[CIT0079] Galvão RM , Li M, Kothadia SM, Haskel JD, Decker PV, Van Buskirk EK, Chen M. 2012. Photoactivated phytochromes interact with HEMERA and promote its accumulation to establish photomorphogenesis in Arabidopsis. Genes & Development 26, 1851–1863. 22895253 10.1101/gad.193219.112 PMC3426763

[CIT0080] Gaudin V , Libault M, Pouteau S, Juul T, Zhao G, Lefebvre D, Grandjean O. 2001. Mutations in LIKE HETEROCHROMATIN PROTEIN 1 affect flowering time and plant architecture in Arabidopsis. Development 128, 4847–4858. 11731464 10.1242/dev.128.23.4847

[CIT0081] Gelato KA , Tauber M, Ong MS, et al. 2014. Accessibility of different histone H3-binding domains of UHRF1 is allosterically regulated by phosphatidylinositol 5-phosphate. Molecular Cell 54, 905–919. 24813945 10.1016/j.molcel.2014.04.004

[CIT0082] Gendall AR , Levy YY, Wilson A, Dean C. 2001. The VERNALIZATION 2 gene mediates the epigenetic regulation of vernalization in Arabidopsis. Cell 107, 525–535. 11719192 10.1016/s0092-8674(01)00573-6

[CIT0083] Gent JI , Wang N, Dawe RK. 2017. Stable centromere positioning in diverse sequence contexts of complex and satellite centromeres of maize and wild relatives. Genome Biology 18, 121. 28637491 10.1186/s13059-017-1249-4 PMC5480163

[CIT0084] Gerasimova TI , Corces VG. 1998. Polycomb and trithorax group proteins mediate the function of a chromatin insulator. Cell 92, 511–521. 9491892 10.1016/s0092-8674(00)80944-7

[CIT0085] Ghave-Helm Y , Jankoswki A, Meiers S, Viales RR, Korbel JO, Furlong EEM. 2019. Highly rearranged chromosomes reveal uncoupling between genome topology and gene expression. Nature Genetics 51, 1272–1282. 31308546 10.1038/s41588-019-0462-3 PMC7116017

[CIT0086] Gibson BA , Doolittle LK, Schneider MWG, et al 2019. The role of 3D genome organization in development and cell differentiation. Cell 179, 470–484.e21.31543265

[CIT0087] Goto C , Tamura K, Fukao Y, Shimada T, Hara-Nishimura I. 2014. The novel nuclear envelope protein KAKU4 modulates nuclear morphology in Arabidopsis. The Plant Cell 26, 2143–2155. 24824484 10.1105/tpc.113.122168 PMC4079374

[CIT0088] Gottschling DE , Aparicio OM, Billington BL, Zakian VA. 1990. Position effect at *S. cerevisiae* telomeres: reversible repression of Pol II transcription. Cell 63, 751–762. 2225075 10.1016/0092-8674(90)90141-z

[CIT0089] Greider CW , Blackburn EH. 1985. Identification of a specific telomere terminal transferase activity in *Tetrahymena* extracts. Cell 43, 405–413. 3907856 10.1016/0092-8674(85)90170-9

[CIT0090] Grob S , Schmid MW, Grossniklaus U. 2014. Hi-C analysis in Arabidopsis identifies the KNOT, a structure with similarities to the flamenco locus of *Drosophila.* Molecular Cell 55, 678–693. 25132176 10.1016/j.molcel.2014.07.009

[CIT0091] Groves NR , Biel AM, Newman-Griffis AH, Meier I. 2018. Dynamic changes in plant nuclear organization in response to environmental and developmental signals. Plant Physiology 176, 230–241. 28739821 10.1104/pp.17.00788 PMC5761808

[CIT0092] Grummt I , Pikaard CS. 2003. Epigenetic silencing of RNA polymerase I transcription. Nature Reviews. Molecular Cell Biology 11, 2124–2136. 10.1038/nrm1171 12923526

[CIT0093] Gumber HH , Joseph FM, Estrada AL, Tolmie AF, Graumann K, Bass HW. 2019. Identification and characterization of genes encoding the nuclear envelope LINC complex in the monocot species *Zea mays*. Journal of Cell Science 132, jcs221390. 30659121 10.1242/jcs.221390

[CIT0094] Guo T , Fang Y. 2014. Functional organization and dynamics of the cell nucleus. Frontiers in Plant Science 5, 378. 25161658 10.3389/fpls.2014.00378 PMC4130368

[CIT0095] Guo T , Mao X, Zhang H, Zhang Y, Fu M, Sun Z, Kuai P, Lou Y, Fang Y. 2017. Lamin-like proteins negatively regulate plant immunity through NAC WITH TRANSMEMBRANE MOTIF1-LIKE9 and NONEXPRESSOR OF PR GENES1 in *Arabidopsis thaliana*. Molecular Plant 10, 1334–1348. 28943325 10.1016/j.molp.2017.09.008

[CIT0096] Hall LL , Smith KP, Byron M, Lawrence JB. 2006. Molecular anatomy of a speckle. The Anatomical Record. Part A, Discoveries in Molecular, Cellular, and Evolutionary Biology 288, 664–675. 10.1002/ar.a.20336 PMC2563428 16761280

[CIT0097] Harris CJ , Husmann D, Liu W, et al. 2016. Arabidopsis AtMORC4 and AtMORC7 form nuclear bodies and repress a large number of protein-coding genes. PLoS Genetics 12, e1005998. 27171361 10.1371/journal.pgen.1005998 PMC4865129

[CIT0098] Hayflick L . 1982. Biological aspects of human ageing. British Journal of Hospital Medicine 27, 366, 368, passim. 7074274

[CIT0099] Heacock M , Spangler E, Riha K, Puizina J, Shippen DE. 2004. Molecular analysis of telomere fusions in Arabidopsis: multiple pathways for chromosome end-joining. The EMBO Journal 23, 2304–2313. 15141167 10.1038/sj.emboj.7600236 PMC419913

[CIT0100] Heitz E . 1931. Nukleolen und Chromosomen in der Gattung *Vicia*. Planta 15, 494–505.

[CIT0101] Hirakawa T , Matsunaga S. 2019. Characterization of DNA repair foci in root cells of arabidopsis in response to DNA damage. Frontiers in Plant Science 10, 990. 31417598 10.3389/fpls.2019.00990 PMC6682680

[CIT0102] Hnisz D , Weintraub AS, Day DS, et al. 2016. Activation of proto-oncogenes by disruption of chromosome neighborhoods. Science 351, 1454–1458. 26940867 10.1126/science.aad9024 PMC4884612

[CIT0103] Hohenstatt ML , Mikulski P, Komarynets O, Klose C, Kycia I, Jeltsch A, Farrona S, Schubert D. 2018. PWWP-DOMAIN INTERACTOR OF POLYCOMBS1 interacts with polycomb-group proteins and histones and regulates Arabidopsis flowering and development. The Plant Cell 30, 117–133. 29330200 10.1105/tpc.17.00117 PMC5810566

[CIT0104] Holec S , Berger F. 2012. Polycomb group complexes mediate developmental transitions in plants. Plant Physiology 158, 35–43. 22086420 10.1104/pp.111.186445 PMC3252096

[CIT0105] Hu B , Wang N, Bi X, Karaaslan ES, Weber AL, Zhu W, Berendzen KW, Liu C. 2019. Plant lamin-like proteins mediate chromatin tethering at the nuclear periphery. Genome Biology 20, 87. 31039799 10.1186/s13059-019-1694-3 PMC6492433

[CIT0106] Huang H , McLoughlin KE, Sorkin ML, Burgie ES, Bindbeutel RK, Vierstra RD, Nusinow DA. 2019. PCH1 regulates light, temperature, and circadian signaling as a structural component of phytochrome B-photobodies in Arabidopsis. Proceedings of the National Academy of Sciences, USA 116, 8603–8608. 10.1073/pnas.1818217116 PMC6486730 30948632

[CIT0107] Huang Y , Jiang L, Liu B-Y, Tan C-F, Chen D-H, Shen W-H, Ruan Y. 2019. Evolution and conservation of polycomb repressive complex 1 core components and putative associated factors in the green lineage. BMC Genomics 20, 533. 31253095 10.1186/s12864-019-5905-9 PMC6599366

[CIT0108] Jacob MD , Audas TE, Uniacke J, Trinkle-Mulcahy L, Lee S. 2013. Environmental cues induce a long noncoding RNA-dependent remodeling of the nucleolus. Molecular Biology of the Cell 24, 2943–2953. 23904269 10.1091/mbc.E13-04-0223 PMC3771955

[CIT0109] Jagannathan M , Cummings R, Yamashita YM. 2019. The modular mechanism of chromocenter formation in *Drosophila*. eLife 8, e43938. 30741633 10.7554/eLife.43938 PMC6382350

[CIT0110] Jagannathan M , Yamashita YM. 2017. Function of junk: pericentromeric satellite DNA in chromosome maintenance. Cold Spring Harbor Symposia on Quantitative Biology 82, 319–327. 29610245 10.1101/sqb.2017.82.034504

[CIT0111] James TC , Elgin SC. 1986. Identification of a nonhistone chromosomal protein associated with heterochromatin in *Drosophila melanogaster* and its gene. Molecular and Cellular Biology 6, 3862–3872. 3099166 10.1128/mcb.6.11.3862-3872.1986 PMC367149

[CIT0112] Kaiserli E , Páldi K, O’Donnell L, Batalov O, Pedmale UV, Nusinow DA, Kay SA, Chory J. 2015. Integration of light and photoperiodic signaling in transcriptional nuclear foci. Developmental Cell 35, 311–321. 26555051 10.1016/j.devcel.2015.10.008 PMC4654455

[CIT0113] Khan A , Garbelli A, Grossi S, et al. 2014. The Arabidopsis STRESS RESPONSE SUPPRESSOR DEAD-box RNA helicases are nucleolar- and chromocenter-localized proteins that undergo stress-mediated relocalization and are involved in epigenetic gene silencing. The Plant Journal 79, 28–43. 24724701 10.1111/tpj.12533

[CIT0114] Kim DH , Sung S. 2014. Polycomb-mediated gene silencing in *Arabidopsis thaliana*. Molecules and Cells 37, 841–850. 25410906 10.14348/molcells.2014.0249 PMC4275700

[CIT0115] Kim J , Han KY, Khanna N, Ha T, Belmont AS. 2019. Nuclear speckle fusion via long-range directional motion regulates speckle morphology after transcriptional inhibition. Journal of Cell Science 132, jcs226563. 30858197 10.1242/jcs.226563 PMC6503955

[CIT0116] Kim W , Ludlow AT, Min J, Robin JD, Stadler G, Mender I, Lai TP, Zhang N, Wright WE, Shay JW. 2016. Regulation of the human telomerase gene TERT by telomere position effect-over long distances (TPE-OLD): implications for aging and cancer. PLoS Biology 14, e2000016. 27977688 10.1371/journal.pbio.2000016 PMC5169358

[CIT0117] Kim W , Shay JW. 2018. Long-range telomere regulation of gene expression: telomere looping and telomere position effect over long distances (TPE-OLD). Differentiation 99, 1–9. 29197683 10.1016/j.diff.2017.11.005 PMC5826875

[CIT0118] Klose C , Viczián A, Kircher S, Schäfer E, Nagy F. 2015. Molecular mechanisms for mediating light-dependent nucleo/cytoplasmic partitioning of phytochrome photoreceptors. New Phytologist 206, 965–971. 10.1111/nph.13207 PMC4406131 26042244

[CIT0119] Koch A , Kang H-G, Steinbrenner J, Dempsey DA, Klessig DF, Kogel K-H. 2017. MORC proteins: novel players in plant and animal health. Frontiers in Plant Science 8, 1720. 29093720 10.3389/fpls.2017.01720 PMC5651269

[CIT0120] Köhler C , Springer N. 2017. Plant epigenomics—deciphering the mechanisms of epigenetic inheritance and plasticity in plants. Genome Biology 18, 132. 28683755 10.1186/s13059-017-1260-9 PMC5501107

[CIT0121] Kotake T , Takada S, Nakahigashi K, Ohto M, Goto K. 2003. Arabidopsis TERMINAL FLOWER 2 gene encodes a heterochromatin protein 1 homolog and represses both FLOWERING LOCUS T to regulate flowering time and several floral homeotic genes. Plant & Cell Physiology 44, 555–564. 12826620 10.1093/pcp/pcg091

[CIT0122] Kotova E , Jarnik M, Tulin AV. 2009. Poly (ADP-ribose) polymerase 1 is required for protein localization to Cajal body. PLoS Genetics 5, e1000387. 19229318 10.1371/journal.pgen.1000387 PMC2637609

[CIT0123] Kovařik A , Fajkus J, Koukalová B, Bezděk M. 1996. Species-specific evolution of telomeric and rDNA repeats in the tobacco composite genome. Theoretical and Applied Genetics 92, 1108–1111. 24166644 10.1007/BF00224057

[CIT0124] Kuchar M , Fajkus J. 2004. Interactions of putative telomere-binding proteins in *Arabidopsis thaliana*: identification of functional TRF2 homolog in plants. FEBS Letters 578, 311–315. 15589838 10.1016/j.febslet.2004.11.021

[CIT0125] Kumaran RI , Thakar R, Spector DL. 2008. Chromatin dynamics and gene positioning. Cell 132, 929–934. 18358806 10.1016/j.cell.2008.03.004 PMC2898133

[CIT0126] Kundu S , Ji F, Sunwoo H, Jain G, Lee JT, Sadreyev RI, Dekker J, Kingston RE. 2017. Polycomb repressive complex 1 generates discrete compacted domains that change during differentiation. Molecular Cell 65, 432–446.e5. 28157505 10.1016/j.molcel.2017.01.009 PMC5421375

[CIT0127] Langdon EM , Gladfelter AS. 2018. A new lens for RNA localization: liquid–liquid phase separation. Annual Review of Microbiology 72, 255–271. 10.1146/annurev-micro-090817-062814 30200855

[CIT0128] Langen G , von Einem S, Koch A, et al. 2014. The compromised recognition of Turnip crinkle virus1 subfamily of microrchidia ATPases regulates disease resistance in barley to biotrophic and necrotrophic pathogens. Plant Physiology 164, 866–878. 24390392 10.1104/pp.113.227488 PMC3912112

[CIT0129] Larson AG , Elnatan D, Keenen MM, Trnka MJ, Johnston JB, Burlingame AL, Agard DA, Redding S, Narlikar GJ. 2017. Liquid droplet formation by HP1α suggests a role for phase separation in heterochromatin. Nature 547, 236–240. 28636604 10.1038/nature22822 PMC5606208

[CIT0130] Lawrence RJ , Earley K, Pontes O, Silva M, Chen ZJ, Neves N, Viegas W, Pikaard CS. 2004. A concerted DNA methylation/histone methylation switch regulates rRNA gene dosage control and nucleolar dominance. Molecular Cell 13, 599–609. 14992728 10.1016/s1097-2765(04)00064-4

[CIT0131] Levy YY , Mesnage S, Mylne JS, Gendall AR, Dean C. 2002. Multiple roles of Arabidopsis VRN1 in vernalization and flowering time control. Science 297, 243–246. 12114624 10.1126/science.1072147

[CIT0132] Lewis AE , Sommer L, Arntzen MØ, Strahm Y, Morrice NA, Divecha N, D’Santos CS. 2011. Identification of nuclear phosphatidylinositol 4,5-bisphosphate-interacting proteins by neomycin extraction. Molecular & Cellular Proteomics 10, M110.003376. 10.1074/mcp.M110.003376 PMC3033679 21048195

[CIT0133] Lewis PW , Elsaesser SJ, Noh KM, Stadler SC, Allis CD. 2010. Daxx is an H3.3-specific histone chaperone and cooperates with ATRX in replication-independent chromatin assembly at telomeres. Proceedings of the National Academy of Sciences, USA 107, 14075–14080. 10.1073/pnas.1008850107 PMC2922592 20651253

[CIT0134] Li HB , Müller M, Bahechar IA, Kyrchanova O, Ohno K, Georgiev P, Pirrotta V. 2011. Insulators, not Polycomb response elements, are required for long-range interactions between Polycomb targets in *Drosophila melanogaster*. Molecular and Cellular Biology 31, 616–625. 21135119 10.1128/MCB.00849-10 PMC3028641

[CIT0135] Li HB , Ohno K, Gui H, Pirrotta V. 2013. Insulators target active genes to transcription factories and polycomb-repressed genes to polycomb bodies. PLoS Genetics 9, e1003436. 23637616 10.1371/journal.pgen.1003436 PMC3630138

[CIT0136] Li P , Banjade S, Cheng HC, et al. 2012. Phase transitions in the assembly of multivalent signalling proteins. Nature 483, 336–340. 22398450 10.1038/nature10879 PMC3343696

[CIT0137] Libault M , Tessadori F, Germann S, Snijder B, Fransz P, Gaudin V. 2005. The Arabidopsis LHP1 protein is a component of euchromatin. Planta 222, 910–925. 16244868 10.1007/s00425-005-0129-4

[CIT0138] Lieberman-Aiden E , van Berkum NL, Williams L, et al. 2009. Comprehensive mapping of long-range interactions reveals folding principles of the human genome. Science 326, 289–293. 19815776 10.1126/science.1181369 PMC2858594

[CIT0139] Lindhout BI , Fransz P, Tessadori F, Meckel T, Hooykaas PJ, van der Zaal BJ. 2007. Live cell imaging of repetitive DNA sequences via GFP-tagged polydactyl zinc finger proteins. Nucleic Acids Research 35, e107. 17704126 10.1093/nar/gkm618 PMC2018617

[CIT0140] Liti G , Louis EJ. 2003. NEJ1 prevents NHEJ-dependent telomere fusions in yeast without telomerase. Molecular Cell 11, 1373–1378. 12769859 10.1016/s1097-2765(03)00177-1

[CIT0141] Liu C , Cheng YJ, Wang JW, Weigel D. 2017. Prominent topologically associated domains differentiate global chromatin packing in rice from Arabidopsis. Nature Plants 3, 742–748. 28848243 10.1038/s41477-017-0005-9

[CIT0142] Liu Q , Shi L, Fang Y. 2012. Dicing bodies. Plant Physiology 158, 61–66. 22025607 10.1104/pp.111.186734 PMC3252078

[CIT0143] Liu X , Wang C, Liu W, et al. 2016. Distinct features of H3K4me3 and H3K27me3 chromatin domains in pre-implantation embryos. Nature 537, 558–562. 27626379 10.1038/nature19362

[CIT0144] Liu Y , Liu Q, Yan Q, Shi L, Fang Y. 2014. Nucleolus-tethering system (NoTS) reveals that assembly of photobodies follows a self-organization model. Molecular Biology of the Cell 25, 1366–1373. 24554768 10.1091/mbc.E13-09-0527 PMC3983000

[CIT0145] Liu YG , Liu H, Chen L, et al. 2002. Development of new transformation-competent artificial chromosome vectors and rice genomic libraries for efficient gene cloning. Gene 282, 247–255. 11814697 10.1016/s0378-1119(01)00849-6

[CIT0146] Liu YG , Shirano Y, Fukaki H, Yanai Y, Tasaka M, Tabata S, Shibata D. 1999. Complementation of plant mutants with large genomic DNA fragments by a transformation-competent artificial chromosome vector accelerates positional cloning. Proceedings of the National Academy of Sciences, USA 96, 6535–6540. 10.1073/pnas.96.11.6535 PMC26917 10339623

[CIT0147] Loubiere V , Martinez AM, Cavalli G. 2019. Cell fate and developmental regulation dynamics by polycomb proteins and 3D genome architecture. BioEssays 41, e1800222. 30793782 10.1002/bies.201800222

[CIT0148] Love AJ , Yu C, Petukhova NV, Kalinina NO, Chen J, Taliansky ME. 2017. Cajal bodies and their role in plant stress and disease responses. RNA Biology 14, 779–790. 27726481 10.1080/15476286.2016.1243650 PMC5519230

[CIT0149] Luke B , Lingner J. 2009. TERRA: telomeric repeat-containing RNA. The EMBO Journal 28, 2503–2510. 19629047 10.1038/emboj.2009.166 PMC2722245

[CIT0150] Lundblad V , Wright WE. 1996. Telomeres and telomerase: a simple picture becomes complex. Cell 87, 369–375. 8898191 10.1016/s0092-8674(00)81358-6

[CIT0151] Lupiáñez DG , Kraft K, Heinrich V, et al. 2015. Disruptions of topological chromatin domains cause pathogenic rewiring of gene–enhancer interactions. Cell 161, 1012–1025. 25959774 10.1016/j.cell.2015.04.004 PMC4791538

[CIT0152] Machyna M , Neugebauer KM, Staněk D. 2015. Coilin: the first 25 years. RNA Biology 12, 590–596. 25970135 10.1080/15476286.2015.1034923 PMC4615369

[CIT0153] Maharana S , Wang J, Papadopoulos DK, et al. 2018. RNA buffers the phase separation behavior of prion-like RNA binding proteins. Science 360, 918–921. 29650702 10.1126/science.aar7366 PMC6091854

[CIT0154] Majerova E , Mandakova T, Vu GT, Fajkus J, Lysak MA, Fojtova M. 2014. Chromatin features of plant telomeric sequences at terminal vs. internal positions. Frontiers inPlant Science 5, 593. 10.3389/fpls.2014.00593 PMC4219495 25408695

[CIT0155] Malik HS , Henikoff S. 2009. Major evolutionary transitions in centromere complexity. Cell 138, 1067–1082. 19766562 10.1016/j.cell.2009.08.036

[CIT0156] Mao YS , Zhang B, Spector DL. 2011. Biogenesis and function of nuclear bodies. Trends in Genetics 27, 295–306. 21680045 10.1016/j.tig.2011.05.006 PMC3144265

[CIT0157] Marian CO , Bordoli SJ, Goltz M, Santarella RA, Jackson LP, Danilevskaya O, Beckstette M, Meeley R, Bass HW. 2003. The maize Single myb histone 1 gene, Smh1, belongs to a novel gene family and encodes a protein that binds telomere DNA repeats in vitro. Plant Physiology 133, 1336–1350. 14576282 10.1104/pp.103.026856 PMC281628

[CIT0158] Masuda K , Xu ZJ, Takahashi S, Ito A, Ono M, Nomura K, Inoue M. 1997. Peripheral framework of carrot cell nucleus contains a novel protein predicted to exhibit a long alpha-helical domain. Experimental Cell Research 232, 173–181. 9141634 10.1006/excr.1997.3531

[CIT0159] Mateo LJ , Murphy SE, Hafner A, Cinquini IS, Walker CA, Boettiger AN. 2019. Visualizing DNA folding and RNA in embryos at single-cell resolution. Nature 568, 49–54. 30886393 10.1038/s41586-019-1035-4 PMC6556380

[CIT0160] Matera AG , Izaguire-Sierra M, Praveen K, Rajendra TK. 2009. Nuclear bodies: random aggregates of sticky proteins or crucibles of macromolecular assembly? Developmental Cell 17, 639–647. 19922869 10.1016/j.devcel.2009.10.017 PMC3101021

[CIT0161] Matheson L , Elderkin S. 2018. Polycomb bodies.In: Lavelle C, Victor JM, eds. Nuclear architecture and dynamics, Vol. 2. Elsevier, 297–320.

[CIT0162] McClintock B . 1934. The relation of a particular chromosomal element to the development of the nucleoli in *Zea mays*. Zeitschrift für Zellforschung und Mikroskopische Anatomie 21, 294–326.

[CIT0163] McClintock B . 1941. The stability of broken ends of chromosomes in *Zea mays*. Genetics 26, 234–282. 17247004 10.1093/genetics/26.2.234 PMC1209127

[CIT0164] McClintock B . 1984. The significance of responses of the genome to challenge. Science 226, 792–801. 15739260 10.1126/science.15739260

[CIT0165] McKittrick E , Gafken PR, Ahmad K, Henikoff S. 2004. Histone H3.3 is enriched in covalent modifications associated with active chromatin. Proceedings of the National Academy of Sciences, USA 101, 1525–1530. 10.1073/pnas.0308092100 PMC341768 14732680

[CIT0166] Meier I , Richards EJ, Evans DE. 2017. Cell biology of the plant nucleus. Annual Review of Plant Biology 68, 139–172. 10.1146/annurev-arplant-042916-041115 28226231

[CIT0167] Melek M , Shippen DE. 1996. Chromosome healing: spontaneous and programmed de novo telomere formation by telomerase. Bioessays 18, 301–308. 8967898 10.1002/bies.950180408

[CIT0168] Mikulski P , Hohenstatt ML, Farrona S, Smaczniak C, Stahl Y, Kalyanikrishna, Kaufmann K, Angenent G, Schubert D. 2019. The chromatin-associated protein PWO1 interacts with plant nuclear lamin-like components to regulate nuclear size. The Plant Cell 31, 1141–1154. 30914470 10.1105/tpc.18.00663 PMC6533023

[CIT0169] Mintz PJ , Spector DL. 2000. Compartmentalization of RNA processing factors within nuclear speckles. Journal of Structural Biology 129, 241–251. 10806074 10.1006/jsbi.2000.4213

[CIT0170] Moissiard G , Cokus SJ, Cary J, et al. 2012. MORC family ATPases required for heterochromatin condensation and gene silencing. Science 336, 1448–1451. 22555433 10.1126/science.1221472 PMC3376212

[CIT0171] Molitor AM , Latrasse D, Zytnicki M, et al. 2016. The Arabidopsis hnRNP-Q protein LIF2 and the PRC1 subunit LHP1 function in concert to regulate the transcription of stress-responsive genes. The Plant Cell 28, 2197–2211. 27495811 10.1105/tpc.16.00244 PMC5059796

[CIT0172] Montacié C , Durut N, Opsomer A, et al. 2017. Nucleolar proteome analysis and proteasomal activity assays reveal a link between nucleolus and 26S proteasome in *A. thaliana*. Frontiers in Plant Science 8, 1815. 29104584 10.3389/fpls.2017.01815 PMC5655116

[CIT0173] Montero JJ , López-Silanes I, Megías D, F Fraga M, Castells-García Á, Blasco MA. 2018. TERRA recruitment of polycomb to telomeres is essential for histone trimethylation marks at telomeric heterochromatin. Nature Communications 9, 1548. 10.1038/s41467-018-03916-3 PMC5906467 29670078

[CIT0174] Moyzis RK , Buckingham JM, Cram LS, Dani M, Deaven LL, Jones MD, Meyne J, Ratliff RL, Wu JR. 1988. A highly conserved repetitive DNA sequence, (TTAGGG)n, present at the telomeres of human chromosomes. Proceedings of the National Academy of Sciences, USA 85, 6622–6626. 10.1073/pnas.85.18.6622 PMC282029 3413114

[CIT0175] Mozgová I , Mikulski P, Pecinka A, Farrona S. 2019. Epigenetic mechanisms of abiotic stress response and memory in plants.In: Alvarez-Venegas R, De-la-Pena C, Cassas-Molano JA, eds. Epigenetics in plants of agronomic importance: fundamentals and applications. Cham: Springer International Publishing, 1–64.

[CIT0176] Nagaki K , Talbert PB, Zhong CX, Dawe RK, Henikoff S, Jiang J. 2003. Chromatin immunoprecipitation reveals that the 180-bp satellite repeat is the key functional DNA element of *Arabidopsis thaliana* centromeres. Genetics 163, 1221–1225. 12663558 10.1093/genetics/163.3.1221 PMC1462492

[CIT0177] Nakamura TM , Moser BA, Russell P. 2002. Telomere binding of checkpoint sensor and DNA repair proteins contributes to maintenance of functional fission yeast telomeres. Genetics 161, 1437–1452. 12196391 10.1093/genetics/161.4.1437 PMC1462227

[CIT0178] Németh A , Conesa A, Santoyo-Lopez J, Medina I, Montaner D, Péterfia B, Solovei I, Cremer T, Dopazo J, Längst G. 2010. Initial genomics of the human nucleolus. PLoS Genetics 6, e1000889. 20361057 10.1371/journal.pgen.1000889 PMC2845662

[CIT0179] Ng CK , Kinoshita T, Pandey S, Shimazaki K, Assmann SM. 2004. Abscisic acid induces rapid subnuclear reorganization in guard cells. Plant Physiology 134, 1327–1331. 15084726 10.1104/pp.103.034728 PMC419809

[CIT0180] Nicodemi M , Pombo A. 2014. Models of chromosome structure. Current Opinion in Cell Biology 28, 90–95. 24804566 10.1016/j.ceb.2014.04.004

[CIT0181] Ohtani M . 2017. Plant snRNP biogenesis: a perspective from the nucleolus and cajal bodies. Frontiers in Plant Science 8, 2184. 29354141 10.3389/fpls.2017.02184 PMC5758608

[CIT0182] Olovnikov AM . 1971. Principle of marginotomy in template synthesis of polynucleotides. Doklady Akademii Nauk SSSR 201, 1496–1499. 5158754

[CIT0183] Ozer O , Hickson ID. 2018. Pathways for maintenance of telomeres and common fragile sites during DNA replication stress. Open Biology 8, 180018. 29695617 10.1098/rsob.180018 PMC5936717

[CIT0184] Padeken J , Mendiburo MJ, Chlamydas S, Schwarz HJ, Kremmer E, Heun P. 2013. The nucleoplasmin homolog NLP mediates centromere clustering and anchoring to the nucleolus. Molecular Cell 50, 236–249. 23562326 10.1016/j.molcel.2013.03.002

[CIT0185] Palm D , Simm S, Darm K, Weis BL, Ruprecht M, Schleiff E, Scharf C. 2016. Proteome distribution between nucleoplasm and nucleolus and its relation to ribosome biogenesis in *Arabidopsis thaliana*. RNA Biology 13, 441–454. 26980300 10.1080/15476286.2016.1154252 PMC5038169

[CIT0186] Pawar V , Poulet A, Détourné G, Tatout C, Vanrobays E, Evans DE, Graumann K. 2016. A novel family of plant nuclear envelope-associated proteins. Journal of Experimental Botany 67, 5699–5710. 27630107 10.1093/jxb/erw332

[CIT0187] Pecinka A , Dinh HQ, Baubec T, Rosa M, Lettner N, Mittelsten Scheid O. 2010. Epigenetic regulation of repetitive elements is attenuated by prolonged heat stress in Arabidopsis. The Plant Cell 22, 3118–3129. 20876829 10.1105/tpc.110.078493 PMC2965555

[CIT0188] Pecinka A , Schubert V, Meister A, Kreth G, Klatte M, Lysak MA, Fuchs J, Schubert I. 2004. Chromosome territory arrangement and homologous pairing in nuclei of *Arabidopsis thaliana* are predominantly random except for NOR-bearing chromosomes. Chromosoma 113, 258–269. 15480725 10.1007/s00412-004-0316-2

[CIT0189] Pederson T . 2011. The nucleolus. Cold Spring Harbor Perspectives in Biology 3, 1–15. 10.1101/cshperspect.a000638 PMC3039934 21106648

[CIT0190] Pendle AF , Clark GP, Boon R, Lewandowska D, Lam YW, Andersen J, Mann M, Lamond AI, Brown JW, Shaw PJ. 2005. Proteomic analysis of the Arabidopsis nucleolus suggests novel nucleolar functions. Molecular Biology of the Cell 16, 260–269. 15496452 10.1091/mbc.E04-09-0791 PMC539170

[CIT0191] Picart C , Pontvianne F. 2017. Plant nucleolar DNA: green light shed on the role of nucleolin in genome organization. Nucleus 8, 11–16. 27644794 10.1080/19491034.2016.1236167 PMC5287095

[CIT0192] Picart-Picolo A , Picart C, Picault N, Pontvianne F. 2020. Nucleolus-associated chromatin domains are maintained under heat stress, despite nucleolar reorganization in *Arabidopsis thaliana*. Journal of Plant Research doi: 10.1007/s10265-020-01201-3. 32372397

[CIT0193] Picart-Picolo A , Picault N, Pontvianne F. 2019. Ribosomal RNA genes shape chromatin domains associating with the nucleolus. Nucleus 10, 67–72. 30870088 10.1080/19491034.2019.1591106 PMC6527388

[CIT0194] Pirrotta V , Li HB. 2012. A view of nuclear polycomb bodies. Current Opinion in Genetics & Development 22, 101–109. 22178420 10.1016/j.gde.2011.11.004 PMC3329586

[CIT0195] Pitchiaya S , Mourao MDA, Jalihal AP, Xiao L, Jiang X, Chinnaiyan AM, Schnell S, Walter NG. 2019. Dynamic recruitment of single RNAs to processing bodies depends on RNA functionality. Molecular Cell 74, 521–533.e6. 30952514 10.1016/j.molcel.2019.03.001 PMC6499680

[CIT0196] Plys AJ , Davis CP, Kim J, Rizki G, Keenen MM, Marr SK, Kingston RE. 2019. Phase separation of Polycomb-repressive complex 1 is governed by a charged disordered region of CBX2. Genes & Development 33, 799–813. 31171700 10.1101/gad.326488.119 PMC6601514

[CIT0197] Pontes O , Pikaard CS. 2008. siRNA and miRNA processing: new functions for Cajal bodies. Current Opinion in Genetics & Development 18, 197–203. 18337083 10.1016/j.gde.2008.01.008 PMC2483300

[CIT0198] Pontes O , Vitins A, Ream TS, Hong E, Pikaard CS, Costa-Nunes P. 2013. Intersection of small RNA pathways in *Arabidopsis thaliana* sub-nuclear domains. PLoS One 8, e65652. 23776518 10.1371/journal.pone.0065652 PMC3680462

[CIT0199] Pontvianne F , Blevins T, Chandrasekhara C, Feng W, Stroud H, Jacobsen SE, Michaels SD, Pikaard CS. 2012. Histone methyltransferases regulating rRNA gene dose and dosage control in Arabidopsis. Genes & Development 26, 945–957. 22549957 10.1101/gad.182865.111 PMC3347792

[CIT0200] Pontvianne F , Blevins T, Chandrasekhara C, et al. 2013. Subnuclear partitioning of rRNA genes between the nucleolus and nucleoplasm reflects alternative epiallelic states. Genes & Development 27, 1545–1550. 23873938 10.1101/gad.221648.113 PMC3731543

[CIT0201] Pontvianne F , Boyer-Clavel M, Sáez-Vásquez J. 2016 *a*. Fluorescence-activated nucleolus sorting in Arabidopsis. Methods in Molecular Biology 1455, 203–211. 27576720 10.1007/978-1-4939-3792-9_15

[CIT0202] Pontvianne F , Carpentier MC, Durut N, et al. 2016 *b*. Identification of nucleolus-associated chromatin domains reveals a role for the nucleolus in 3D organization of the *A. thaliana* genome. Cell Reports 16, 1574–1587. 27477271 10.1016/j.celrep.2016.07.016 PMC5279810

[CIT0203] Pontvianne F , Grob S. 2020. Three-dimensional nuclear organization in *Arabidopsis thaliana*. Journal of Plant Research doi: 10.1007/s10265-020-01185-0. 32240449

[CIT0204] Pontvianne F , Liu C. 2019. Chromatin domains in space and their functional implications. Current Opinion in Plant Biology 54, 1–10. 31881292 10.1016/j.pbi.2019.11.005

[CIT0205] Pontvianne F , Matía I, Douet J, Tourmente S, Medina FJ, Echeverria M, Sáez-Vásquez J. 2007. Characterization of AtNUC-L1 reveals a central role of nucleolin in nucleolus organization and silencing of AtNUC-L2 gene in Arabidopsis. Molecular Biology of the Cell 18, 369–379. 17108323 10.1091/mbc.E06-08-0751 PMC1783796

[CIT0206] Pott S , Lieb JD. 2015. What are super-enhancers? Nature Genetics 47, 8–12. 25547603 10.1038/ng.3167

[CIT0207] Poulet A , Duc C, Voisin M, Desset S, Tutois S, Vanrobays E, Benoit M, Evans DE, Probst AV, Tatout C. 2017 *a*. The LINC complex contributes to heterochromatin organisation and transcriptional gene silencing in plants. Journal of Cell Science 130, 590–601. 28049722 10.1242/jcs.194712

[CIT0208] Poulet A , Probst AV, Graumann K, Tatout C, Evans D. 2017 *b*. Exploring the evolution of the proteins of the plant nuclear envelope. Nucleus 8, 46–59. 27644504 10.1080/19491034.2016.1236166 PMC5287204

[CIT0209] Probst AV , Mittelsten Scheid O. 2015. Stress-induced structural changes in plant chromatin. Current Opinion in Plant Biology 27, 8–16. 26042538 10.1016/j.pbi.2015.05.011

[CIT0210] Qiu Y , Pasoreck EK, Reddy AK, Nagatani A, Ma W, Chory J, Chen M. 2017. Mechanism of early light signaling by the carboxy-terminal output module of Arabidopsis phytochrome B. Nature Communications 8, 1905. 10.1038/s41467-017-02062-6 PMC5712524 29199270

[CIT0211] Quinodoz SA , Ollikainen N, Tabak B, et al. 2018. Higher-order inter-chromosomal hubs shape 3D genome organization in the nucleus. Cell 174, 744–757.e24. 29887377 10.1016/j.cell.2018.05.024 PMC6548320

[CIT0212] Reddy AS , Day IS, Göhring J, Barta A. 2012. Localization and dynamics of nuclear speckles in plants. Plant Physiology 158, 67–77. 22045923 10.1104/pp.111.186700 PMC3252098

[CIT0213] Ren X , Vincenz C, Kerppola TK. 2008. Changes in the distributions and dynamics of polycomb repressive complexes during embryonic stem cell differentiation. Molecular and Cellular Biology 28, 2884–2895. 18316406 10.1128/MCB.00949-07 PMC2293085

[CIT0214] Riera M , Redko Y, Leung J. 2006. Arabidopsis RNA-binding protein UBA2a relocalizes into nuclear speckles in response to abscisic acid. FEBS Letters 580, 4160–4165. 16828085 10.1016/j.febslet.2006.06.064

[CIT0215] Richards EJ , Ausubel FM. 1988. Isolation of a higher eukaryotic telomere from *Arabidopsis thaliana*. Cell 53, 127–136. 3349525 10.1016/0092-8674(88)90494-1

[CIT0216] Ritossa FM , Spiegelman S. 1965. Localization of DNA complementary to ribosomal RNA in the nucleolus organizer region of *Drosophila melanogaster*. Proceedings of the National Academy of Sciences, USA 53, 737–745. 10.1073/pnas.53.4.737 PMC221060 14324529

[CIT0217] Rosa S , De Lucia F, Mylne JS, Zhu D, Ohmido N, Pendle A, Kato N, Shaw P, Dean C. 2013. Physical clustering of FLC alleles during Polycomb-mediated epigenetic silencing in vernalization. Genes & Development 27, 1845–1850. 24013499 10.1101/gad.221713.113 PMC3778238

[CIT0218] Rosa S , Shaw P. 2013. Insights into chromatin structure and dynamics in plants. Biology 2, 1378–1410. 24833230 10.3390/biology2041378 PMC4009787

[CIT0219] Sabari BR , Dall’Agnese A, Boija A, et al 2018. Coactivator condensation at super-enhancers links phase separation and gene control. Science 361, eaar3958. 10.1126/science.aar3958 PMC6092193 29930091

[CIT0220] Sáez-Vásquez J , Delseny M. 2019. Ribosome biogenesis in plants: from functional 45S ribosomal DNA organization to ribosome assembly factors. The Plant Cell 31, 1945–1967. 31239391 10.1105/tpc.18.00874 PMC6751116

[CIT0221] Sakamoto Y , Takagi S. 2013. LITTLE NUCLEI 1 and 4 regulate nuclear morphology in *Arabidopsis thaliana*. Plant & Cell Physiology 54, 622–633. 23396599 10.1093/pcp/pct031

[CIT0222] Sanborn AL , Rao SS, Huang SC, et al. 2015. Chromatin extrusion explains key features of loop and domain formation in wild-type and engineered genomes. Proceedings of the National Academy of Sciences, USA 112, E6456–E6465. 10.1073/pnas.1518552112 PMC4664323 26499245

[CIT0223] Santos AP , Abranches R, Oliveira M, Shaw P. 2015. Plasticity of chromatin organization in the plant interphase nucleus. In: Pontes O, Jin H, eds. Nuclear functions in plant transcription, signaling and development. New York: Springer New York, 57–79.

[CIT0224] Santos AP , Abranches R, Stoger E, Beven A, Viegas W, Shaw PJ. 2002. The architecture of interphase chromosomes and gene positioning are altered by changes in DNA methylation and histone acetylation. Journal of Cell Science 115, 4597–4605. 12415004 10.1242/jcs.00160

[CIT0225] Santos AP , Ferreira LJ, Oliveira MM. 2017. Concerted flexibility of chromatin structure, methylome, and histone modifications along with plant stress responses. Biology 6, 3. 10.3390/biology6010003 PMC5371996 28275209

[CIT0226] Santos AP , Shaw P. 2004. Interphase chromosomes and the Rabl configuration: does genome size matter? Journal of Microscopy 214, 201–206. 15102067 10.1111/j.0022-2720.2004.01324.x

[CIT0227] Sawyer IA , Sturgill D, Sung MH, Hager GL, Dundr M. 2016. Cajal body function in genome organization and transcriptome diversity. BioEssays 38, 1197–1208. 27767214 10.1002/bies.201600144 PMC5225948

[CIT0228] Scarpin R , Sigaut L, Pietrasanta L, McCormick S, Zheng B, Muschietti J. 2013. Cajal bodies are developmentally regulated during pollen development and pollen tube growth in *Arabidopsis thaliana*. Molecular Plant 6, 1355–1357. 23699706 10.1093/mp/sst077

[CIT0229] Schoeftner S , Blasco MA. 2008. Developmentally regulated transcription of mammalian telomeres by DNA-dependent RNA polymerase II. Nature Cell Biology 10, 228–236. 18157120 10.1038/ncb1685

[CIT0230] Schořová Š , Fajkus J, Záveská Drábková L, Honys D, Schrumpfová PP. 2019. The plant Pontin and Reptin homologues, RuvBL1 and RuvBL2a, colocalize with TERT and TRB proteins in vivo, and participate in telomerase biogenesis. The Plant Journal 98, 195–212. 30834599 10.1111/tpj.14306

[CIT0231] Schrumpfová PP , Vychodilová I, Dvořáčková M, Majerská J, Dokládal L, Schořová S, Fajkus J. 2014. Telomere repeat binding proteins are functional components of Arabidopsis telomeres and interact with telomerase. The Plant Journal 77, 770–781. 24397874 10.1111/tpj.12428 PMC4282523

[CIT0232] Schubert V , Weisshart K. 2015. Abundance and distribution of RNA polymerase II in Arabidopsis interphase nuclei. Journal of Experimental Botany 66, 1687–1698. 25740920 10.1093/jxb/erv091 PMC4357323

[CIT0233] Schwarzacher T , Anamthawat-Jónsson K, Harrison GE, et al. 1992. Genomic in situ hybridization to identify alien chromosomes and chromosome segments in wheat. Theoretical and Applied Genetics 84, 778–786. 24201474 10.1007/BF00227384

[CIT0234] Sequeira-Mendes J , Aragüez I, Peiró R, Mendez-Giraldez R, Zhang X, Jacobsen SE, Bastolla U, Gutierrez C. 2014. The functional topography of the Arabidopsis genome is organized in a reduced number of linear motifs of chromatin states. The Plant Cell 26, 2351–2366. 24934173 10.1105/tpc.114.124578 PMC4114938

[CIT0235] Shah FR , Bhat YA, Wani AH. 2018. Subnuclear distribution of proteins: links with genome architecture. Nucleus 9, 42–55. 28910577 10.1080/19491034.2017.1361578 PMC5973252

[CIT0236] Shaw J , Love AJ, Makarova SS, Kalinina NO, Harrison BD, Taliansky ME. 2014. Coilin, the signature protein of Cajal bodies, differentially modulates the interactions of plants with viruses in widely different taxa. Nucleus 5, 85–94. 24637832 10.4161/nucl.28315 PMC4028359

[CIT0237] Shaw PJ , Brown JW. 2004. Plant nuclear bodies. Current Opinion in Plant Biology 7, 614–620. 15491908 10.1016/j.pbi.2004.09.011

[CIT0238] Shibata F , Murata M. 2004. Differential localization of the centromere-specific proteins in the major centromeric satellite of *Arabidopsis thaliana*. Journal of Cell Science 117, 2963–2970. 15161939 10.1242/jcs.01144

[CIT0239] Shin Y , Chang YC, Lee DSW, Berry J, Sanders DW, Ronceray P, Wingreen NS, Haataja M, Brangwynne CP. 2018. Liquid nuclear condensates mechanically sense and restructure the genome. Cell 175, 1481–1491.e13. 30500535 10.1016/j.cell.2018.10.057 PMC6724728

[CIT0240] Simon L , Voisin M, Tatout C, Probst AV. 2015. Structure and function of centromeric and pericentromeric heterochromatin in *Arabidopsis thaliana*. Frontiers in Plant Science 6, 1049. 26648952 10.3389/fpls.2015.01049 PMC4663263

[CIT0241] Smythe C , Jenkins HE, Hutchison CJ. 2000. Incorporation of the nuclear pore basket protein nup153 into nuclear pore structures is dependent upon lamina assembly: evidence from cell-free extracts of *Xenopus* eggs. The EMBO Journal 19, 3918–3931. 10921874 10.1093/emboj/19.15.3918 PMC306609

[CIT0242] Snoek BL , Pavlova P, Tessadori F, Peeters AJM, Bourbousse C, Barneche F, de Jong H, Fransz PF, van Zanten M. 2017. Genetic dissection of morphometric traits reveals that phytochrome B affects nucleus size and heterochromatin organization in *Arabidopsis thaliana*. G3 7, 2519–2531. 28592555 10.1534/g3.117.043539 PMC5555459

[CIT0243] Sobol M , Krausová A, Yildirim S, et al 2018. Nuclear phosphatidylinositol 4,5-bisphosphate islets contribute to efficient RNA polymerase II-dependent transcription. Journal of Cell Science 131, jcs.211094. 10.1242/jcs.211094 29507116

[CIT0244] Soppe WJ , Jasencakova Z, Houben A, Kakutani T, Meister A, Huang MS, Jacobsen SE, Schubert I, Fransz PF. 2002. DNA methylation controls histone H3 lysine 9 methylation and heterochromatin assembly in Arabidopsis. The EMBO Journal 21, 6549–6559. 12456661 10.1093/emboj/cdf657 PMC136960

[CIT0245] Sotelo-Silveira M , Chávez Montes RA, Sotelo-Silveira JR, Marsch-Martínez N, de Folter S. 2018. Entering the next dimension: plant genomes in 3D. Trends in Plant Science 23, 598–612. 29703667 10.1016/j.tplants.2018.03.014

[CIT0246] Spector DL , Lamond AI. 2011. Nuclear speckles. Cold Spring Harbor Perspectives in Biology 3, 1–12. 10.1101/cshperspect.a000646 PMC3039535 20926517

[CIT0247] Stam M , Tark-Dame M, Fransz P. 2019. 3D genome organization: a role for phase separation and loop extrusion? Current Opinion in Plant Biology 48, 36–46. 31035031 10.1016/j.pbi.2019.03.008

[CIT0248] Staněk D , Fox AH. 2017. Nuclear bodies: news insights into structure and function. Current Opinion in Cell Biology 46, 94–101. 28577509 10.1016/j.ceb.2017.05.001

[CIT0249] Stępiński D . 2014. Functional ultrastructure of the plant nucleolus. Protoplasma 251, 1285–1306. 24756369 10.1007/s00709-014-0648-6 PMC4209244

[CIT0250] Strom AR , Brangwynne CP. 2019. The liquid nucleome—phase transitions in the nucleus at a glance. Journal of Cell Science 132, jcs.235093. 10.1242/jcs.235093 PMC6899023 31754043

[CIT0251] Strom AR , Emelyanov AV, Mir M, Fyodorov DV, Darzacq X, Karpen GH. 2017. Phase separation drives heterochromatin domain formation. Nature 547, 241–245. 28636597 10.1038/nature22989 PMC6022742

[CIT0252] Szabo Q , Bantignies F, Cavalli G. 2019. Principles of genome folding into topologically associating domains. Science Advances 5, eaaw1668. 30989119 10.1126/sciadv.aaw1668 PMC6457944

[CIT0253] Szabo Q , Jost D, Chang JM, et al. 2018. TADs are 3D structural units of higher-order chromosome organization in Drosophila. Science Advances 4, eaar8082. 29503869 10.1126/sciadv.aar8082 PMC5829972

[CIT0254] Sztacho M , Sobol M, Balaban C, Escudeiro Lopes SE, Hozák P. 2019. Nuclear phosphoinositides and phase separation: important players in nuclear compartmentalization. Advances in Biological Regulation 71, 111–117. 30249540 10.1016/j.jbior.2018.09.009

[CIT0255] Tamura K , Fukao Y, Iwamoto M, Haraguchi T, Hara-Nishimura I. 2010. Identification and characterization of nuclear pore complex components in *Arabidopsis thaliana*. The Plant Cell 22, 4084–4097. 21189294 10.1105/tpc.110.079947 PMC3027183

[CIT0256] Tamura K , Hara-Nishimura I. 2011. Involvement of the nuclear pore complex in morphology of the plant nucleus. Nucleus 2, 168–172. 21818409 10.4161/nucl.2.3.16175 PMC3149876

[CIT0257] Tatavosian R , Kent S, Brown K, Yao T, Duc HN, Huynh TN, Zhen CY, Ma B, Wang H, Ren X. 2019. Nuclear condensates of the Polycomb protein chromobox 2 (CBX2) assemble through phase separation. Journal of Biological Chemistry 294, 1451–1463. 10.1074/jbc.RA118.006620 PMC6364756 30514760

[CIT0258] Tek AL , Kashihara K, Murata M, Nagaki K. 2010. Functional centromeres in soybean include two distinct tandem repeats and a retrotransposon. Chromosome Research 18, 337–347. 20204495 10.1007/s10577-010-9119-x

[CIT0259] Tek AL , Kashihara K, Murata M, Nagaki K. 2011. Functional centromeres in *Astragalus sinicus* include a compact centromere-specific histone H3 and a 20-bp tandem repeat. Chromosome Research 19, 969–978. 22065151 10.1007/s10577-011-9247-y

[CIT0260] Turck F , Roudier F, Farrona S, Martin-Magniette ML, Guillaume E, Buisine N, Gagnot S, Martienssen RA, Coupland G, Colot V. 2007. Arabidopsis TFL2/LHP1 specifically associates with genes marked by trimethylation of histone H3 lysine 27. PLoS Genetics 3, e86. 17542647 10.1371/journal.pgen.0030086 PMC1885283

[CIT0261] Ukmar-Godec T , Hutten S, Grieshop MP, Rezaei-Ghaleh N, Cima-Omori MS, Biernat J, Mandelkow E, Söding J, Dormann D, Zweckstetter M. 2019. Lysine/RNA-interactions drive and regulate biomolecular condensation. Nature Communications 10, 2909. 10.1038/s41467-019-10792-y PMC6606616 31266957

[CIT0262] Van Buskirk EK , Decker PV, Chen M. 2012. Photobodies in light signaling. Plant Physiology 158, 52–60. 21951469 10.1104/pp.111.186411 PMC3252093

[CIT0263] Van Buskirk EK , Reddy AK, Nagatani A, Chen M. 2014. Photobody localization of phytochrome B is tightly correlated with prolonged and light-dependent inhibition of hypocotyl elongation in the dark. Plant Physiology 165, 595–607. 24769533 10.1104/pp.114.236661 PMC4044834

[CIT0264] van Koningsbruggen S , Gierlinski M, Schofield P, Martin D, Barton GJ, Ariyurek Y, den Dunnen JT, Lamond AI. 2010. High-resolution whole-genome sequencing reveals that specific chromatin domains from most human chromosomes associate with nucleoli. Molecular Biology of the Cell 21, 3735–3748. 20826608 10.1091/mbc.E10-06-0508 PMC2965689

[CIT0265] Vaquero-Sedas MI , Luo C, Vega-Palas MA. 2012. Analysis of the epigenetic status of telomeres by using ChIP-seq data. Nucleic Acids Research 40, e163. 22855559 10.1093/nar/gks730 PMC3505975

[CIT0266] Vaquero-Sedas MI , Vega-Palas MA. 2013. Differential association of Arabidopsis telomeres and centromeres with histone H3 variants. Scientific Reports 3, 1202. 23383372 10.1038/srep01202 PMC3563029

[CIT0267] Vertii A , Ou J, Yu J, Yan A, Pagès H, Liu H, Zhu LJ, Kaufman PD. 2019. Two contrasting classes of nucleolus-associated domains in mouse fibroblast heterochromatin. Genome Research 29, 1235–1249. 31201210 10.1101/gr.247072.118 PMC6673712

[CIT0268] Vrbsky J , Akimcheva S, Watson JM, Turner TL, Daxinger L, Vyskot B, Aufsatz W, Riha K. 2010. siRNA-mediated methylation of Arabidopsis telomeres. PLoS Genetics 6, e1000986. 20548962 10.1371/journal.pgen.1000986 PMC2883606

[CIT0269] Walker MP , Tian L, Matera AG. 2009. Reduced viability, fertility and fecundity in mice lacking the cajal body marker protein, coilin. PLoS One 4, e6171. 19587784 10.1371/journal.pone.0006171 PMC2702818

[CIT0270] Wang H , Dittmer TA, Richards EJ. 2013. Arabidopsis CROWDED NUCLEI (CRWN) proteins are required for nuclear size control and heterochromatin organization. BMC Plant Biology 13, 200. 24308514 10.1186/1471-2229-13-200 PMC3922879

[CIT0271] Wang L , Gao Y, Zheng X, et al. 2019. Histone modifications regulate chromatin compartmentalization by contributing to a phase separation mechanism. Molecular Cell 76, 646–659. 31543422 10.1016/j.molcel.2019.08.019

[CIT0272] Wang M , Wang P, Lin M, Ye Z, Li G, Tu L, Shen C, Li J, Yang Q, Zhang X. 2018. Evolutionary dynamics of 3D genome architecture following polyploidization in cotton. Nature Plants 4, 90–97. 29379149 10.1038/s41477-017-0096-3

[CIT0273] Wang Q , Sawyer IA, Sung MH, Sturgill D, Shevtsov SP, Pegoraro G, Hakim O, Baek S, Hager GL, Dundr M. 2016. Cajal bodies are linked to genome conformation. Nature Communications 7, 10966. 10.1038/ncomms10966 PMC4802181 26997247

[CIT0274] Wang RC , Smogorzewska A, de Lange T. 2004. Homologous recombination generates T-loop-sized deletions at human telomeres. Cell 119, 355–368. 15507207 10.1016/j.cell.2004.10.011

[CIT0275] Warner JR , Soeiro R. 1967. Nascent ribosomes from HeLa cells. Proceedings of the National Academy of Sciences, USA 58, 1984–1990. 10.1073/pnas.58.5.1984 PMC223894 5237493

[CIT0276] Weaver DT . 1998. Telomeres: moonlighting by DNA repair proteins. Current Biology 8, R492–R494. 9663383 10.1016/s0960-9822(98)70315-x

[CIT0277] West JA , Mito M, Kurosaka S, et al. 2016. Structural, super-resolution microscopy analysis of paraspeckle nuclear body organization. Journal of Cell Biology 214, 817–830. 10.1083/jcb.201601071 PMC5037409 27646274

[CIT0278] White MR , Mitrea DM, Zhang P, Stanley CB, Cassidy DE, Nourse A, Phillips AH, Tolbert M, Taylor JP, Kriwacki RW. 2019. C9orf72 Poly(PR) dipeptide repeats disturb biomolecular phase separation and disrupt nucleolar function. Molecular Cell 74, 713–728.e6. 30981631 10.1016/j.molcel.2019.03.019 PMC6525025

[CIT0279] Xin R , Kathare PK, Huq E. 2019. Coordinated regulation of pre-mRNA splicing by the SFPS–RRC1 complex to promote photomorphogenesis. The Plant Cell 31, 2052–2069. 31266850 10.1105/tpc.18.00786 PMC6751115

[CIT0280] Xin R , Zhu L, Salomé PA, Mancini E, Marshall CM, Harmon FG, Yanovsky MJ, Weigel D, Huq E. 2017. SPF45-related splicing factor for phytochrome signaling promotes photomorphogenesis by regulating pre-mRNA splicing in Arabidopsis. Proceedings of the National Academy of Sciences, USA 114, E7018–E7027. 10.1073/pnas.1706379114 PMC5565451 28760995

[CIT0281] Yao RW , Xu G, Wang Y, et al. 2019. Nascent pre-rRNA sorting via phase separation drives the assembly of dense fibrillar components in the human nucleolus. Molecular Cell 76, 767–783. 31540874 10.1016/j.molcel.2019.08.014

[CIT0282] Yildirim S , Castano E, Sobol M, Philimonenko VV, Dzijak R, Venit T, Hozák P. 2013. Involvement of phosphatidylinositol 4,5-bisphosphate in RNA polymerase I transcription. Journal of Cell Science 126, 2730–2739. 23591814 10.1242/jcs.123661

[CIT0283] Yokoyama Y , Takahashi Y, Shinohara A, Lian Z, Xiaoyun W, Niwa K, Tamaya T. 1998. Telomerase activity is found in the epithelial cells but not in the stromal cells in human endometrial cell culture. Molecular Human Reproduction 4, 985–989. 9809681 10.1093/molehr/4.10.985

[CIT0284] Yoo CY , Pasoreck EK, Wang H, Cao J, Blaha GM, Weigel D, Chen M. 2019. Phytochrome activates the plastid-encoded RNA polymerase for chloroplast biogenesis via nucleus-to-plastid signaling. Nature Communications 10, 2629. 10.1038/s41467-019-10518-0 PMC6570650 31201355

[CIT0285] Zambrano-Mila MS , Aldaz-Villao MJ, Armando Casas-Mollano J. 2019. Canonical histones and their variants in plants: evolution and functions.In: Alvarez-Venegas R, De-la-Pena C, Cassas-Molano JA, eds. Epigenetics in plants of agronomic importance: fundamentals and applications. Cham: Springer International Publishing, 185–222.

[CIT0286] Zhang C , Du X, Tang K, et al. 2018. Arabidopsis AGDP1 links H3K9me2 to DNA methylation in heterochromatin. Nature Communications 9, 4547. 10.1038/s41467-018-06965-w PMC6208443 30382101

[CIT0287] Zhang W , Lee HR, Koo DH, Jiang J. 2008. Epigenetic modification of centromeric chromatin: hypomethylation of DNA sequences in the CENH3-associated chromatin in *Arabidopsis thaliana* and maize. The Plant Cell 20, 25–34. 18239133 10.1105/tpc.107.057083 PMC2254920

[CIT0288] Zhang X , Germann S, Blus BJ, Khorasanizadeh S, Gaudin V, Jacobsen SE. 2007. The Arabidopsis LHP1 protein colocalizes with histone H3 Lys27 trimethylation. Nature Structural & Molecular Biology 14, 869–871. 10.1038/nsmb1283 17676062

[CIT0289] Zhang Y , Bertulat B, Tencer AH, Ren X, Wright GM, Black J, Cardoso MC, Kutateladze TG. 2019. MORC3 forms nuclear condensates through phase separation. iScience 17, 182–189. 31284181 10.1016/j.isci.2019.06.030 PMC6614601

[CIT0290] Zhao S , Cheng L, Gao Y, Zhang B, Zheng X, Wang L, Li P, Sun Q, Li H. 2019. Plant HP1 protein ADCP1 links multivalent H3K9 methylation readout to heterochromatin formation. Cell Research 29, 54–66. 30425322 10.1038/s41422-018-0104-9 PMC6318295

[CIT0291] Zheng H , Xie W. 2019. The role of 3D genome organization in development and cell differentiation. Nature Reviews. Molecular Cell Biology 20, 535–550. 31197269 10.1038/s41580-019-0132-4

[CIT0292] Zhou H , Song Z, Zhong S, Zuo L, Qi Z, Qu LJ, Lai L. 2019. Mechanism of DNA-induced phase separation for transcriptional repressor VRN1. Angewandte Chemie 58, 4858–4862. 30762296 10.1002/anie.201810373

[CIT0293] Zhou Y , Hartwig B, James GV, Schneeberger K, Turck F. 2016. Complementary activities of TELOMERE REPEAT BINDING proteins and polycomb group complexes in transcriptional regulation of target genes. The Plant Cell 28, 87–101. 26721861 10.1105/tpc.15.00787 PMC4746681

[CIT0294] Zhou Y , Wang Y, Krause K, Yang T, Dongus JA, Zhang Y, Turck F. 2018. Telobox motifs recruit CLF/SWN–PRC2 for H3K27me3 deposition via TRB factors in Arabidopsis. Nature Genetics 50, 638–644. 29700471 10.1038/s41588-018-0109-9

[CIT0295] Zhu L , Brangwynne CP. 2015. Nuclear bodies: the emerging biophysics of nucleoplasmic phases. Current Opinion in Cell Biology 34, 23–30. 25942753 10.1016/j.ceb.2015.04.003 PMC5562147

