# Corrigendum to: Tidying-up the plant nuclear space: domains, functions, and dynamics

**DOI:** 10.1093/jxb/erab134

**Published:** 2021-07-01

**Authors:** Ana Paula Santos, Valérie Gaudin, Iva Mozgová, Frédéric Pontvianne, Daniel Schubert, Ahmet L Tek, Martina Dvořáčková, Chang Liu, Paul Fransz, Stefanie Rosa, Sara Farrona

**Affiliations:** 1 Instituto de Tecnologia Química e Biológica António Xavier, Universidade Nova de Lisboa, Oeiras, Portugal; 2 Institut Jean-Pierre Bourgin, INRAE, AgroParisTech, Université Paris-Saclay, 78000, Versailles, France; 3 Biology Centre of the Czech Academy of Sciences, České Budějovice, Czech Republic; 4 Faculty of Science, University of South Bohemia, České Budějovice, Czech Republic; 5 CNRS, Laboratoire Génome et Développement des Plantes (LGDP), Université de Perpignan Via Domitia, Perpignan, France; 6 Institute for Biology, Freie Universität Berlin, Berlin, Germany; 7 Agricultural Genetic Engineering Department, Niğde Ömer Halisdemir University, Niğde, Turkey; 8 CEITEC/Masaryk University, Brno, Czech Republic; 9 Center for Plant Molecular Biology (ZMBP), University of Tübingen, Tübingen, Germany; 10 Institute of Biology, University of Hohenheim, Stuttgart, Germany; 11 University of Amsterdam, Amsterdam, The Netherlands; 12 Swedish University of Agricultural Sciences, Uppsala, Sweden; 13 Plant and AgriBiosciences Centre, Ryan Institute, NUI Galway, Galway, Ireland

*Journal of Experimental Botany*, Volume 71, Issue 17, 17 August 2020, Pages 5160–5178, doi:10.1093/jxb/eraa282

This article has been corrected to include a missing funding declaration in the acknowledgements section and to make an amendment to one other funding source in the same section. No other changes have been made.

